# Serotonin distinctly controls behavioral states in restrained and freely moving *Drosophila*

**DOI:** 10.1016/j.isci.2022.105886

**Published:** 2022-12-28

**Authors:** Swetha B.M. Gowda, Ayesha Banu, Safa Salim, Kadir A. Peker, Farhan Mohammad

**Affiliations:** 1 Division of Biological and Biomedical Sciences (BBS), College of Health & Life Sciences (CHLS), Hamad Bin Khalifa University (HBKU), Doha 34110, Qatar; 2 Pegasystems Inc., Istanbul, Turkey

**Keywords:** Behavioral neuroscience, Molecular neuroscience, Cellular neuroscience

## Abstract

When trapped in a physical restraint, animals must select an escape strategy to increase their chances of survival. After falling into an inescapable trap, they react with stereotypical behaviors that differ from those displayed in escapable situations. Such behaviors involve either a wriggling response to unlock the trap or feigning death to fend off a predator attack. The neural mechanisms that regulate animal behaviors have been well characterized for escapable situations but not for inescapable traps. We report that restrained vinegar flies exhibit alternating flailing and immobility to free themselves from the trap. We used optogenetics and intersectional genetic approaches to show that, while broader serotonin activation promotes immobility, serotonergic cells in the ventral nerve cord (VNC) regulate immobility states majorly via 5-HT7 receptors. Restrained and freely moving locomotor states are controlled by distinct mechanisms. Taken together, our study has identified serotonergic switches of the VNC that promote environment-specific adaptive behaviors.

## Highlights

• Restrained *Drosophila* uses alternating states of flailing and immobility
• Multiple factors distinctly affect behaviors in restraint and freely moving *Drosophila*
• 5-HT^VNC^ distinctly affects behaviors in restraint and freely moving *Drosophila*
• *Drosophila* uses 5-HT7 receptors to enhance immobility states

## Graphical abstract

**Figure:**
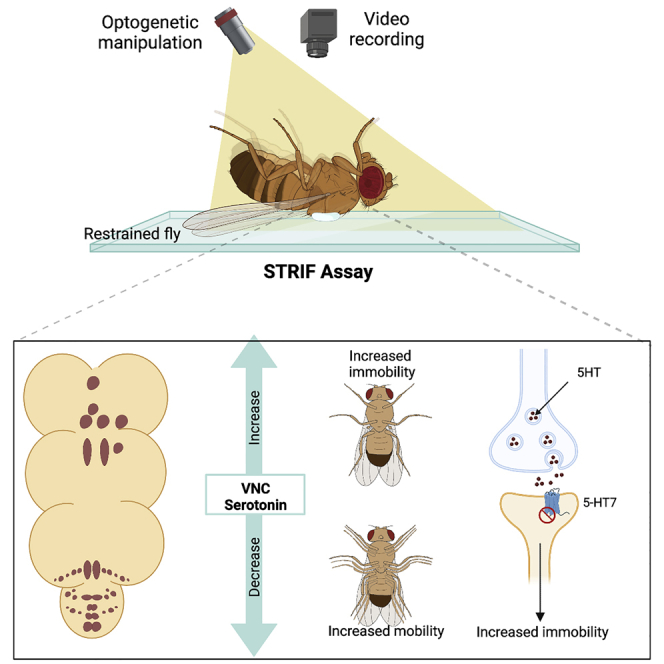

## Introduction

Successful escape from restraining traps is essential for all animals as failure to escape can lead to death. Animals can respond to danger in several ways. Trapped or restrained animals exhibit behaviors that mainly reflect the interplay between two types of motor responses: a state of flailing or wriggling, bursts of high activity, and periods of behavioral quiescence or arrest.^1^^,^^2^ These behaviors have been described in vertebrates and invertebrates, including insects.^3^^,^^4^^,^^5^^,^^6^^,^^7^

Factors such as the proximity and intensity of the threat, escape chances, and predator or trap fitness may determine the decision of the restrained animal to wriggle or arrest movement.^1^^,^^2^^,^^8^^,^^9^^,^^10^^,^^11^ Wriggling, or active struggle, signals the animal’s physical fitness, which is a strategy to unlock the trap. Behavioral arrest, or sustained immobility, is a bold defensive strategy that a restrained prey can use when in peril. While a wriggling response can trigger a further attack from the predator, a temporary behavioral arrest may reduce the predator’s aggression and loosen its grip, increasing the prey’s chances of escaping instead of becoming food.

In humans, behavioral arrest, or tonic immobility, may be linked to episodes of paralysis during traumatic events.^12^^,^^13^^,^^14^ Emerging evidence suggests a strong correlation between trauma-induced tonic immobility and post-traumatic stress disorder (PTSD).^15^^,^^16^ Therefore, understanding how animals respond to threats and how their nervous system reacts to the state of restraint would help determine how serious threats are processed in animals and how emotional behaviors are encoded by the nervous system, both at the level of specific neural circuits and distinct subtypes of neurons that regulate them. Given the evolutionary prevalence of this behavior, it is critical to study it in an experimental setting and define its general governing principles.

In low and medium threat-like situations, such as being trapped in an enclosed environment, a freely moving *Drosophila* exhibits anxiety-like and fear-like (freezing) behaviors.^5^^,^^17^ To study the behavioral response of *Drosophila* in a high-threat situation like an inescapable trap, we developed a robust assay called Struggle Response in Immobilized Fly (STRIF), in which the activity and immobility responses of the restrained flies were recorded and analyzed. The STRIF assay can reproduce an ethologically relevant situation, such as an insect caught in a spider web or the nectar of a carnivorous plant. In this paradigm, the restrained *Drosophila* makes various escape attempts, mainly alternating states of flailing and immobility. The STRIF assay provides a valuable model for understanding the neural mechanisms that control the biphasic expression of oppositional behavioral states. Owing to its high-throughput nature combined with automated analysis, we can quickly test many flies.

Neuromodulatory serotonin mediates signals from all areas of the nervous system representing the animal’s sensory environment and internal states.^18^ Serotonin contextualizes sensory information by physiological and external states, modulating motor outputs by reconfiguring neural properties.^19^ Furthermore, serotonin is implicated in restraint-induced tonic immobility.^2^^,^^20^^,^^21^^,^^22^ However, the neural pathways and mechanisms that control the states of flailing and immobility in restrained animals remain unknown. Using optogenetics and intersectional genetics, in the present study, we demonstrate that optogenetic activation and inactivation in broader serotonin neurons enhance immobility and flailing states, respectively. In these restrained flies, the serotonergic neurons in the ventral nerve cord (VNC) regulate these behavioral states. In addition, we also show that serotonin mediates behavioral responses through 5-HT7 serotonin receptors in the VNC. Using a regression analysis approach, we show that serotonin has varied effects on behavioral output in restrained flies compared to freely moving flies. Changes in locomotor parameters obtained from potential factors, optogenetic interventions, and serotonergic receptors in freely moving *Drosophila*, could not explain variance obtained in changes in STRIF parameters in a restrained state, suggesting that independent mechanisms largely govern these two behavioral states. Our results suggest that the serotonergic system is critical in the flies’ escape struggle behaviors in inescapable trap-like situations. Moreover, they indicate that multiple serotonin pathways regulate different aspects of escape struggle in a trap-like situation, helping fruit flies to escape effectively. Taken together, *Drosophila* fruit flies represent a suitable model for studying the neural mechanisms underlying defensive behaviors in inescapable situations.

## Results

### A restrained fly exhibits a variety of escape struggle behaviors

**Figure 1:**
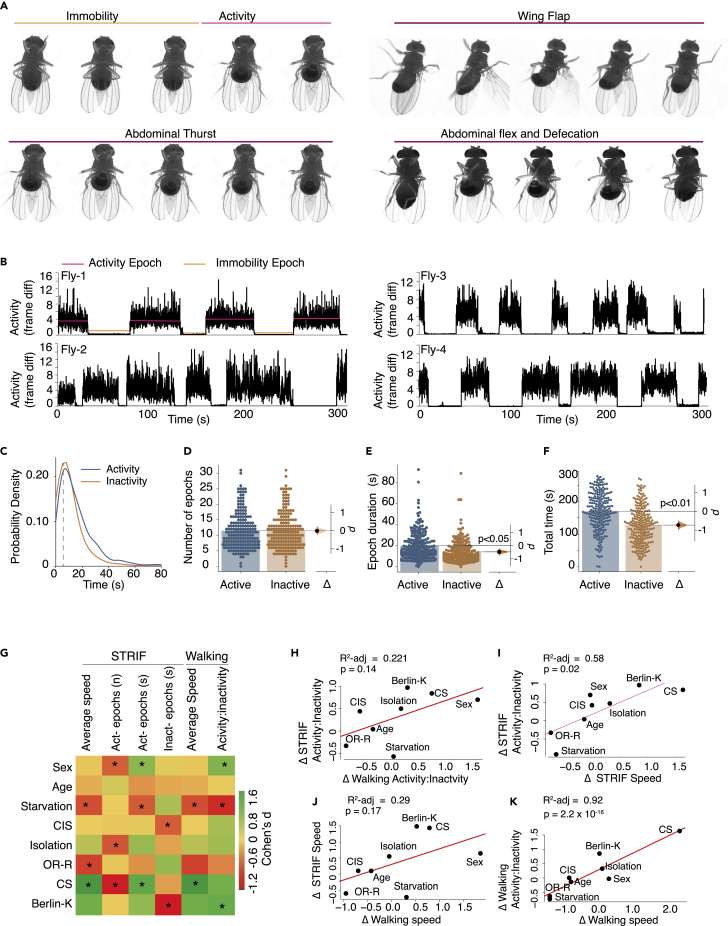
Struggle Response in Immobilized Fly (STRIF) Assay (A) Muybridge images representing various escape-like behaviors of wild-type (*w*^*1118*^) fruit flies in a restrained state. (B) Representative locomotor traces show the states of activity and inactivity throughout the duration of the assay (300 s). (C) Probabilistic distribution of activity and inactivity durations with 5s bin, dashed line depicts 5s (n-flies = 54, total 1,824 periods were analyzed for activity and inactivity time. (D) Restrained flies show a similar number of activity and inactivity epochs during the assay (300 s), (n = 198, Δ = −0.01, *d* = −0.01 [95CI −0.21, 0.2], p *= 0.93)*. (E) The average duration of activity and inactivity epochs during the assay (300 s). Flies spent significantly more time in activity epochs than inactivity epochs (n = 200, Δ = −0.43, *d* = −0.47 [95CI −0.66, −0.25], p *= 0.0001)*. (F) The average total time in the activity and inactivity state during the assay (300 s), flies spent significantly more time in activity than in inactivity (n = 218, Δ = −0.72, *d* = −0.73 [95CI −0.93, −0.53], p *= 0.0001)*. Each dot on the scatterplots represents the data of one fly. Cohen’s *d* represents the effect size. The p *value* was obtained using a two-sided permutation t-test. The *n* denotes the number of flies used in each experiment. (G) The heatmap shows various ΔSTRIF and Δwalking parameters for various physiological factors. Plotted values represent the Cohen’s *d* effect size for each intervention. The values marked with asterisks represent statistically significant effect sizes between the control and experimental animals (p < 0.05). (H) Linear regression model of locomotor activity-inactivity ratios depicted as a predictor of STRIF activity-inactivity ratios—for eight manipulations obtained from the analysis of STRIF assay (Figures S1A–S1F) and walking parameters in an open field assay (Figures S2A and S2B), and the locomotor activity-inactivity ratios were not predictive of the STRIF activity-inactivity ratios. (I) STRIF speed is moderately predictive of STRIF activity-inactivity ratios. (J) Linear regression model of STRIF activity was also not predictive of locomotor activity. (K) Linear regression model of locomotor speed indicates the locomotor speed as a predictor of STRIF speed. See also Figures S1 and S2, and Videos S1, S2, S3, S4, S5, S6, S7, S8, and S9. CIS: chronic immobilization stress.

To reproduce the restrained state in a laboratory setting, we glued flies to their thorax on a glass slide. In this state, a fly can make body movements using the abdomen, legs, and wings in a bid to escape, which we named STRIF behavior. To study flies’ STRIF behavior, we assayed six flies simultaneously, video-recorded their activity, and analyzed them using a python script developed in-house. We recorded the activity of individual flies for 5 minutes (Video S1). In our experiment, restrained fruit flies made various efforts to escape: locomotor efforts consisted of alternating periods of flailing (an escape struggle with a burst of high activity) and immobility (the state of quiescence or inactivity) (Figures 1A and 1B). Restrained flies also exhibited abdominal thrusts (Figure 1A, Videos S1, S2, S3, S4, S5, S6, S7, S8, and S9), anterior and posterior grooming, abdominal flexion, wing flapping (Videos S1, S2, S3, S4, S5, S6, S7, S8, and S9), and defecation (Figure 1A). Each episode of activity or inactivity captured the movement of the legs, abdomen, and wings. By analyzing the movements of individual restrained flies, we measured the duration and frequency of some of these states. The inactivity duration of the flies was the most frequent between 5 and 20 second (Figure 1C). We used a minimum of 5 second as the threshold for measuring immobility. Specifically, our metrics were the average time spent in activity (STRIF-activity) and inactivity (STRIF-immobility), the number and duration of activity and inactivity epochs, and average speed of activity epochs. Although restrained male wild-type (*w*^*1118*^) flies had an equal number of activity and inactivity epochs (n = 198, Δ = −0.01, *d* = −0.01 [95CI −0.21, 0.2], p *= 0.0001)* (Figures 1B and 1D), active epochs lasted longer in duration than inactivity epochs (n = 200, Δ = −0.43, *d* = −0.47 [95CI −0.66, −0.25], p *= 0.0001)* (Figure 1E). Overall, the flies spent significantly more time in STRIF-activity (n = 218, Δ = −0.72, *d* = −0.73 [95CI −0.93, −0.53], p *= 0.0001)* (Figure 1F) during the assay time.

Video S1. Video showing activity vs inactivity in STRIF assay, related to Figure 1

Video S2. Video showing activity vs inactivity in STRIF assay, related to Figure 1

Video S3. Video showing activity vs inactivity and abdominal thrusts in STRIF assay, related to Figure 1

Video S4. Video showing activity vs inactivity and abdominal thrusts in STRIF assay, related to Figure 1

Video S5. Video showing activity vs inactivity and abdominal thrusts in STRIF assay, related to Figure 1

Video S6. Video showing activity vs inactivity and abdominal thrusts in STRIF assay, related to Figure 1

Video S7. Video showing wing flapping in STRIF assay, related to Figure 1

Video S8. Video showing wing movement in STRIF assay, related to Figure 1

Video S9. Video showing upside down activity vs inactivity in STRIF assay, related to Figure 1

### Potential factors influencing the STRIF response of a fly in a restrained state

To facilitate an effective escape, the animal’s brain must quickly integrate all the necessary information from the dynamic environment to minimize reaction time and maximize the chances of escape when encountering a predator or trap. However, many environmental factors, such as the nature of the threat, genetic background, past experiences, sex, age, and internal state (e.g., hunger and anxiety), may influence an animal’s defensive response. Consequently, we studied the potential factors that may affect the fly’s struggle to escape from a restrained state, including sex (male vs. female), physiological status (fed vs. starved, age, immobility), and genetic background (*Canton-S [CS], w*^*1118*^*, Oregon-R [OR-R],* and *Berlin-K*).

We tested the level of escape struggle in males and females and found that STRIF-based activity-inactivity ratios were significantly higher in restrained females than in males (n = 36–37, Δ = 1.05, *d* = 0.7 [95CI 0.24, 1.1], p *= 0.006)* (Figures 1G and S1A). Moreover, female flies had a significantly higher number but shorter duration of activity epochs, while the average flailing speed of activity epochs and the duration of inactivity epochs remained the same as in males (Figure 1G). This suggests that escape struggle, quantified using the STRIF paradigm, induces a relatively general motivation to escape with higher reactivity in females. Physiological conditions, such as age or satiety, can influence the driving force of behavior by modulating the sensory system processing or acting on neuromodulators like serotonin.^23^ We found that age did not affect any parameter of the STRIF response (n = 53, Δ = 0.04, d = 0.04 [95CI −0.34, 0.43], p *= 0.83)* (Figures 1G and S1B). At the same time, 24 h of starvation decreased the STRIF-based activity-inactivity ratios and the average flailing speed (n = 47–53, Δ = 0.50, d = −0.58 [95CI −0.92, −0.077], p *= 0.003)* (Figures 1G and S1C) but increased the duration of inactivity epochs compared to fed flies (Figure 1G). Taken together, this data suggests that in the STRIF assay, escape struggle was resistant to age but was affected by satiety.

Immobility and social isolation have previously been shown to affect anxiety and stress-related behaviors in rodents and flies.^5^^,^^17^ Compared with normal flies, flies that were chronically immobilized for three days (6 h each day) showed slightly increased STRIF activity-inactivity ratios and shorter inactivity epochs (n = 36–44, Δ = 0.72, *d* = 0.42 [95CI −0.031, 0.79], p *= 0.052*) (Figures 1G and S1D). We then assayed the STRIF response of flies socially isolated for seven days and compared their STRIF response to flies in a group with other flies. Socially isolated flies also showed an enhanced STRIF activity-inactivity ratio (n = 40–41, Δ = 0.47, *d* = 0.47 [95CI 0.006, 0.96], p *= 0.03*) (Figure S1E) and increased duration of activity epochs (Figure 1G); however, the number of activity epochs was lower (Figure 1G). Overall, our data suggest that in *Drosophila*, the motivation to struggle was reinforced by past negative experiences, such as immobility and social isolation.

Next, we studied the influence of the genetic background on escape struggle, which was measured in common laboratory strains (*CS, OR-R,* and *Berlin-K*) using the STRIF paradigm. While all strains showed a higher level of STRIF-activity over STRIF-immobility (Figure S1F), there were differences in STRIF response levels between the strains. While *CS* and *Berlin-K* fly exhibited strong escape responses, *OR-R* and *w*^*1118*^ flies exhibited medium-level struggle responses compared to *w*^*1118*^ isogenic flies (n = 34–42, *w*^*1118*^ -*OR-R*, Δ = −0.28, d = −0.31, [95CI −0.68, 0.259], p = 0.17; *w*^*1118*^ -*CS*, Δ = 1.0, d = 0.85, [95CI 0.248, 1.33], p = 0.0006; *w*^*1118*^ -*Berlin-K*, Δ = 1.11, d = 0.98, [95CI 0.34, 1.47], p = 0.0001) (Figure S1F). These experiments suggest that although the escape struggle response in *Drosophila* is evolutionarily conserved, genetic background nevertheless influences its manifestation.

### Fly STRIF motor output is distinct from the locomotor behavior in freely moving conditions

Activity and immobility periods in restrained flies resemble walking and resting periods in freely walking flies. We used the *Drosophila* arousal threshold system^24^ to study whether the behavioral output in restrained flies was similar to the locomotor output in freely moving flies in response to various manipulations (Figures S2A, and S2B). We obtained the ratio of locomotor activity-inactivity and measured the speed of flies placed in an open field arena for a duration of 5 min. We employed a linear regression approach to compare animals’ behavioral output in the open field with behavior in the STRIF assay, asking whether changes in activity-inactivity ratios in freely moving animals could predict the activity-inactivity ratios in restrained animals. Regression analysis of the scores of changes in locomotor activity-inactivity ratios and changes in STRIF activity-inactivity ratios showed that the scores of freely moving flies were not predictive of STRIF activity-inactivity ratios of restrained flies (Figure 1H, *R*^*2*^*adj = 0.22,* p *= 0.14*). We next performed a regression between changes in locomotor speed and STRIF speed. The linear regression model showed that changes in locomotor speed were largely predictive of STRIF speed (Figure 1I*, R*^*2*^*adj = 0.92,* p *= 2.2 x 10*^*−16*^). A linear regression analysis of changes in STRIF activity of restrained flies was also not predictive of locomotor activity in freely moving flies (Figure 1J, *R*^*2*^*adj = 0.29,* p *= 0.17*). Additional regression analyses of STRIF speed changes and STRIF activity-inactivity ratios indicated that STRIF speed could explain only half of the changes in STRIF activity-inactivity ratios (Figure 1K*, R*^*2*^*adj = 0.58,* p *= 0.02*). These results suggest that STRIF activity-inactivity ratios are distinct from the locomotor output, while locomotor speed and STRIF speed are related. Thus, while the speed changes contribute to the changes in STRIF activity-inactivity ratios, they are not the primary driving force of STRIF behavior. Because the correlation between freely moving and restrained flies' activity-inactivity ratios was very small and statistically insignificant, we further studied activity-inactivity ratios as an STRIF behavior.

### Broad serotonergic activity evokes a state of immobility in restrained Drosophila

**Figure 2:**
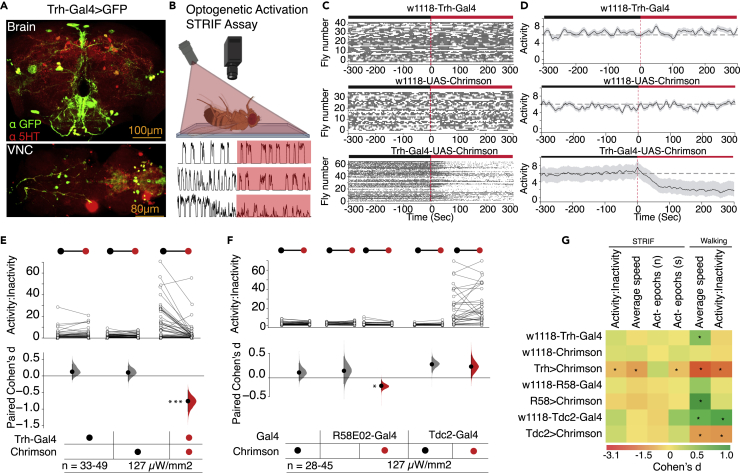
Optogenetic activity in the broader serotonin system promotes the state of inactivity in restrained fruit flies (A) Immunohistochemistry images of serotonin clusters in the brain and VNC stained with α-5-HT (red) and α-GFP (green) in flies expressing mCD8:GFP in *Trh-Gal4*-labeled serotonin cells, scale bar: 100 μm, 80 μm. (B) Schematic depicting the optogenetic STRIF assay used for optogenetic activation of serotonergic neurons in restrained flies using red light illumination that is kept off for the first 5 min and switched on for the next 5 min. Panels below show fly activity measured in this paradigm. (C) Raster plots illustrating the occurrence of STRIF activity and inactivity before and after red illumination (represented by black and red bars respectively); the onset of red illumination is depicted as time = 0 s. (D) Flies expressing Chrimson in *Trh-Gal4* cells showed a strong reduction in activity under red illumination. (E) Red illumination with Chrimson evoked the state of immobility in *Trh-Gal4*>*Chrimson* flies (n = 52, Δ = −0.63, d = −0.806, [95.0% CI -1.08, −0.49], p *= 0.000*1) and a nonsignificant effect on genotypic controls (*w*^*1118*^-*Trh-Gal4* and *w*^*1118*^-*Chrimson*). (F) Optogenetic activation of dopaminergic (*R58E02*-*Gal4*>*Chrimson*, n = 45, Δ = 0.70, *d* = 0.34, [95.0% CI -0.44, −0.18], p *= 0.01*) or octopaminergic neurons (*Tdc2-*Gal4>*Chrimson*, n = 33, Δ = 0.16, *d* = 0.16, [95.0% CI -0.12, 0.48], p *= 0.3*) in the restrained flies resulted in STRIF-activity like that of control flies. The results in panels (E) and (F) denote a paired comparison of before (dark) vs. after (red) illumination of the same fly. The circles correspond to dark and red light illumination. The filled curves of Cohen’s *d* for paired samples in the lower part of panels (E) and (F) show the distribution of the mean difference. The vertical line illustrates the error bar- 95CI of the difference in the filled curves. n represents the sample size. (G) The heatmap shows various ΔSTRIF and Δwalking parameters (related to Figures 2E and 2F). Plotted values represent Cohen’s *d* effect size for each STRIF and walking paradigms intervention. The values marked with asterisks represent statistically significant effect sizes between the control and experimental animals (p < 0.05). Statistical analysis: Paired *t*-test. Two-sided permutation t-test-based p values are displayed as ∗ for p < 0.05, ∗∗ for p < 0.01, and ∗∗∗ for p < 0.001. Keys indicate the genotypes at the top of each panel in (A), (B), (C), and (D), at the bottom in (E) and (F). Optogenetic activation experiments were conducted using 127 μW/mm^2^ red light.

Serotonin influences diverse brain functions, including walking,^25^ circadian entrainment,^26^ sleep,^27^ aggression,^28^ and olfactory learning and memory.^29^ Serotonin also influences various stress-related behaviors in vertebrates and invertebrates, including emotional responses such as anxiety, fear, and panic.^17^^,^^30^^,^^31^ We hypothesized that the overall influence of serotonergic transmission would affect the state of the fly’s brain in a negative-valence trap-like situation. We used red-illumination-sensitive channelrhodopsin (Chrimson) to optogenetically activate^32^ serotonergic or 5-hydroxytryptaminergic (5-HT) neurons under red light (127 μW/mm^2^) (Figures 2A and 2B). Activation of neurotransmission in the majority of *tryptophan hydroxylase* (*Trh*)-bearing serotonergic cells labeled with *Trh-Gal4*^33^ produced a marked decrease in activity compared to their activity before exposure to red illumination (Figures 2C and 2D). Red illumination enhanced the state of immobility in Trh-Gal4>Chrimson flies as STRIF activity-inactivity ratio (n = 52, Δ = −0.63, d = −0.806, [95.0% CI -1.08, −0.49], p = 0.0001), average speed, and duration of activity epochs were significantly reduced (Figures 2E, 2G, and S3A) without affecting numbers of activity epochs (Figures 2G and S3D). The red illumination did not affect the ΔSTRIF response in the controls (*w*^*1118*^-*Trh-Gal4 and w*^*1118*^*-Chrimson*, Figures 2C–2E). Although we saw gender differences in STRIF response in wild-type flies, optogenetic activation of Trh-Gal4 cells showed no gender differences (data not shown). For better comparison with the existing body of literature, where experiments have been performed exclusively in males,^34^ we used male flies in our further experiments. We also evaluated the effect of optogenetic activation of most dopaminergic and octopaminergic/tyraminergic neurons on STRIF behavior. We did not observe any change in STRIF-activity when octopaminergic (*Tdc2*) neurons were optogenetically activated and observed very little change when dopaminergic (*R58E02*) neurons were activated in the restrained flies (Figures 2F and 2G). We further analyzed optogenetic activity-mediated changes in walking speed and activity-inactivity ratios in freely moving flies for serotonergic, octopaminergic, and dopaminergic neurons. While optogenetic activity in serotonergic cells consistently reduces speed and activity-inactivity ratios in STRIF and walking paradigms, activity in the dopaminergic and octopaminergic systems shows distinct effects in STRIF and walking paradigms in the direction or the magnitude of the change. These results are consistent with the idea that acute serotonergic activity promotes the state of immobility and further support the idea that STRIF-response is a distinct response in restrained flies.

### Inhibition of the serotonergic system enhances the *Drosophila* STRIF-activity response

**Figure 3:**
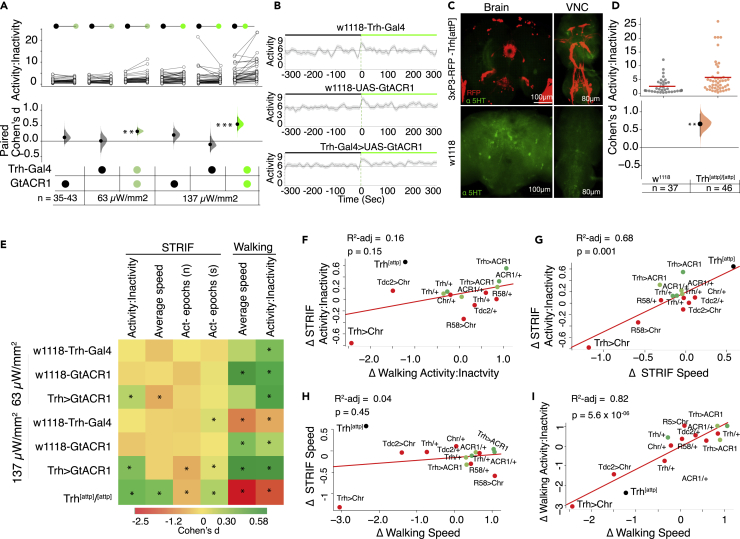
Silencing serotonin neurons increases STRIF-activity (A) Optogenetic inactivation of serotonergic neurons using GtACR1 and green light illumination (*Trh-Gal4>GtACR1)* enhanced the activity state in the restrained flies at both lower green light intensity (63 μW/mm^2^, n = 38, Δ = 0.47, *d* = 0.32, [95.0% CI 0.2, 0.43], p *= 0.0004*) and full green light intensity (137 μW/mm^2^, n = 41, Δ = 0.75, *d* = 0.55, [95.0% CI 0.35, 0.78], p *= 0.0001)*. No effect of either intensity of green light illumination was observed in controls (*w*^*1118*^-*Trh-Gal4, w*^*1118*^*-GtACR1*). Filled curves of paired samples in the lower part of the panels indicate distribution of the mean differences. The vertical line illustrates the error bar- 95CI of the difference in the filled curves. n represents the sample size. (B) Line-plot depicting activity before and after optogenetic inactivation of serotonin neurons using full green light illumination. (C) Immunohistochemistry images of the *Trh*^*[attP]*^ mutant stained with α-5HT (green). 3XP3-RFP is part of a construct swapped with the *Trh* gene and may represent 5-HT expression driven by the hsp70 minimal promoter, mainly active in the eyes. Significant reduction in 5-HT staining is observed in mutant strains compared to control flies. Scale bar: 100 μm, 80 μm. (D) Homozygous *Trh*^*[attP*]^ flies showed higher activity-inactivity ratios compared to *w*^*1118*^ isogenic flies (n = 37–46, Δ = 1.21, *d* = 0.66, [95.0% CI 0.32, 0.95], p *= 0.002*), each dot on the scatterplot represents the STRIF response of one fly, red horizontal line represents mean values of the scatterplot. Two-sided permutation t-test p values are depicted as ∗. The bottom panel displays the experiment-control differences as the effect size (Cohen' s *d*) with an error bar - 95 CI of the difference in the filled curve. (E) Heatmap of changes in STRIF and walking parameters in reference to panels A and D. Plotted values represent Cohen’s *d* effect size for each STRIF and walking paradigms intervention. The values marked with asterisks represent statistically significant effect sizes between the control and experimental animals (p < 0.05). (F) Linear regression model of locomotor speed depicted as a predictor of STRIF speed—for five optogenetic manipulations and genotypic controls, obtained from the analysis of STRIF assay (Figures 2 and 3), and walking parameters in an open field assay (Figures S3A–S3F) show that the locomotor speed was not predictive of the STRIF speed. Red represents optogenetic activation experiments, and green as optogenetic inhibition experiments. (G) Similar to speed, changes in locomotor activity-inactivity ratios were not predictive of changes in STRIF activity-inactivity ratios. (H) Changes in STRIF speed were a modest predictor of changes in STRIF activity-inactivity ratios. (I) Changes in locomotor activity-inactivity ratios were a strong predictor of changes in locomotor speed. Statistical analysis: Paired *t*-test. Two-sided permutation t-test-based p values are displayed as ∗ for p < 0.05, ∗∗ for p < 0.01, and ∗∗∗ for p < 0.001.

While our optogenetic activation experiments determined that acute serotonin activation was sufficient to enhance the immobility state, they did not establish that serotonin was necessary for the modulation of *Drosophila*’s struggle behavior when in a restrained state. We addressed this question by expressing GtACR1, an optogenetic inhibitor of neuronal activity,^35^ in broad serotonergic neurons using two illumination intensities (Figure 3A). While lower green light illumination (63 μW/mm^2^) slightly but significantly enhanced the STRIF-activity state (n = 38, Δ = 0.47, d = 0.32, [95.0% CI 0.2, 0.43], p = 0.0004), STRIF-activity state was modest at higher green light illumination (137 μW/mm^2^, n = 41, Δ = 0.75, *d* = 0.55, [95.0% CI 0.35, 0.78], p *= 0.0001)* (Figure 3B). Green illumination at both applied intensities on restrained flies did not affect STRIF-activity in any of the genotypic controls (*w*^*1118*^-*Trh-Gal4)* (Figures 3B and 3C).

To understand the effect of serotonin chronic depletion on STRIF-activity, we analyzed the behavior of *Trh* mutant flies (*Trh*^*[attP]*^*)*—an insertion mutant deficient in serotonin.^36^ We studied the behavior of *Trh*^*[attP]*^ mutants in homozygous flies and compared their behavior with *w*^*1118*^ isogenic flies. *Trh*^*[attP]*^*/Trh*^*[attP]*^ flies had complete serotonin deficiency in the brain and VNC (Figure 3C). The homozygous (*Trh*^*[attP]*^*/Trh*^*[attP]*^*)* mutants showed increased STRIF-activity (n = 37–46, Δ = 1.21, *d* = 0.66, [95.0% CI 0.32, 0.95], p *= 0.002*) as compared to wild-type flies (Figure 3D), a behavior similar to that of animals with optogenetically silenced 5-HT neurons (Figure 3B). These results are consistent with the idea that serotonin is sufficient and necessary for modulating the fly’s STRIF response in a restrained state.

To study the effect of serotonin activity and inactivity on other STRIF variables, we analyzed average flailing speed and the number of activity epochs in both serotonin-deficient *Trh*^*[attP]*^ mutant flies and through optogenetic serotonin manipulations. Interestingly, although average speed was significantly reduced in flies with optogenetically activated serotonin (Figure 3F), there was a slight reduction in speed in flies with inactivated serotonin at a lower intensity; however, at higher intensity, there was no effect on speed (Figures 3E and S3B). Homozygous *Trh*^*[attP]*^ mutants showed increased average speed compared to wild-type control flies (Figures 3F and S3C). Similarly, we did not observe a difference in the number of activity epochs and duration of activity epochs when the serotonin system was optogenetically activated (Figure 2F). However, optogenetically silenced flies showed significantly smaller activity epochs than control flies (Figures 3F and S3F). Interestingly, homozygous *Trh*^*[attP]*^ mutants did not significantly differ from controls in the number of activity epochs (Figures 3F and S3F). To further confirm the dissociation between locomotor and STRIF motor outputs in the optogenetic paradigm, we regressed the speed and activity-inactivity ratios obtained from locomotor and STRIF behaviors. As shown earlier (Figures 1H–1K), we observed a weak cross-correlation between locomotor and STRIF activity-inactivity ratios and STRIF speed (Figures 3F and 3H). However, within the STRIF assay, speed and activity-inactivity ratios were modest predictors of each other; changes in STRIF activity-inactivity ratios explain 68% of changes in STRIF speed (Figure 3G). Additional regression analysis of fly walking speed changes (delta speed) indicated that these could explain almost 82% of walking activity-inactivity ratio variance (3I).

These results suggest that optogenetic STRIF response depends on acute 5-HT, and effects on average speed and the number of activity epochs vary and may depend on the 5-HT levels.

### Serotonin in VNC cells plays a major role in controlling the STRIF response

**Figure 4:**
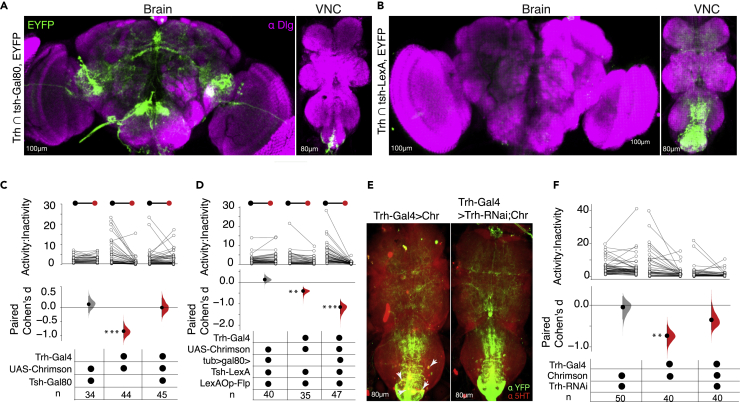
Serotonin in VNC neurons regulates activity and immobility states in restrained flies (A and B) Maximum intensity projections show expression patterns driven by the *Trh-Gal4* labeling serotonergic neurons either in the brain (A), *Trh-Gal4* intersected with *tsh-gal80,* or VNC (B), *Trh-Gal4* genetically crossed with (*tub > gal80>; tsh-LexA, LexAop-Flp; Chrimson*), scale bar: 100 μm, 80 μm. (C) In comparison to optogenetic activation of broad serotonergic neurons (*Trh-Gal4>Chrimson,* n = 44, Δ = −0.63, *d* = −0.84, [95.0% CI -1.1, −0.5], p *= 0.00001*), and genotypic control (*tsh-gal80;Chrimson > w1118),* optogenetic activation of serotonergic neurons in the brain abolished the STRIF response (*Trh* ∩ *tsh-gal80;Chrimson,* n = 45, Δ = −0.001, *d* = −0.001, [95.0% CI -0.33, 0.34], p *= 0.99*). (D) Optogenetic activation of serotonergic neurons in the VNC (*Trh* ∩ *tub > gal80>; tsh-LexA, LexAop-Flp; Chrimson,* n = 47, Δ = −0.83, *d* = −1.15, [95.0% CI -1.46, −0.79], p *= 0.00001*) showed enhanced STRIF-immobility compared to the effect of broader serotonin system activation (*Trh -Chrimson,* n = 35, Δ = −0.32, *d* = −0.5, [95.0% CI -0.59, −0.21], p *= 0.001*) and genotypic control (*tub > gal80>; tsh-LexA, LexAop-Flp; Chrimson > w1118)*. Filled curves of paired samples in the lower part of the panels indicate distribution of the mean differences. The vertical line illustrates the error bar- 95CI of the difference in the filled curves. n represents the sample size. (E) VNC (left panel) from *Trh-Gal4>Chrimson* adult flies with serotonergic clusters stained with anti-5-HT. VNC (right panel) from *Trh-Gal4>Chrimson* adult flies co-expressing *Trh-RNAi1* counterstained with anti-5-HT, scale bar: 80 μm. White arrows indicate GFP co-localization with 5-HT immunostaining in controls. (F) Expression of *Trh-RNAi1* in *Trh-Gal4* cells reduced Chrimson-induced STRIF-immobility (*Trh > Chrimson,* n = 39, Δ = −0.54 *d*ays = −0.72, [95.0% CI -1.0, −0.41], p *= 0.0001),* (*Trh > Trh-RNAi1; Chrimson,* n = 40, Δ = −0.27, *d* = −0.35, [95.0% CI -0.59, 0.07], p *= 0.13)*.

To evaluate whether serotonergic modulation projecting from the brain or VNC is involved in regulating the STRIF response, we used an intersectional genetics approach to limit the expression of serotonin neurons to the brain or VNC by expressing either *tsh-Gal80* in VNC (to inhibit *Trh*-*Gal4* expression in VNC) or Gal80 in the brain using flippase base system (*tub > gal80>; tsh-LexA, LexAop-Flp*) to inhibit expression of *Trh*-*Gal4* in the brain (Figures 4A and 4B). We found that activation of serotonergic neurons intersected in the VNC (*Trh*∩ *tub > gal80>; tsh-LexA,LexAop-Flp;Chrimson,* n = 47, Δ = −0.83, d = −1.15, [95.0% CI -1.46, −0.79], p = 0.00001) but not the brain (*Trh* ∩ *tsh-gal80;Chrimson,* (n = 45, Δ = −0.001, d = −0.001, [95.0% CI -0.33, 0.34], p = 0.99) significantly enhanced the fly’s STRIF-immobility in a restrained state (Figures 4C and 4D). These results suggest that STRIF-activity and immobility are mediated by clusters of serotonergic cells expressed in the VNC.

### STRIF behaviors depend on the serotonin system

We next aimed to determine whether the STRIF response elicited by Trh-Gal4-labeled cells was dependent on serotonin. We depleted serotonin in *Trh* cells using RNAi against TRH, the enzyme essential for synthesizing serotonin. In *Trh-Gal4>Trh-RNAi* flies, immunohistochemistry staining of the serotonin cells showed that *Trh* expression (using anti-5-HT) was drastically reduced (Figure 4E); however, flies with reduced serotonin in the broader *Trh* cells showed immobility that was reduced by 51% (Figure 4F), corresponding to a partial rescue in behavior. Our results suggest that serotonin-independent mechanisms could mediate the *Trh-Gal4*-induced STRIF response. These results suggest that serotonin plays a major role in STRIF behaviors.

### 5-HT7 receptors have a major effect on *Drosophila* STRIF response

**Figure 5:**
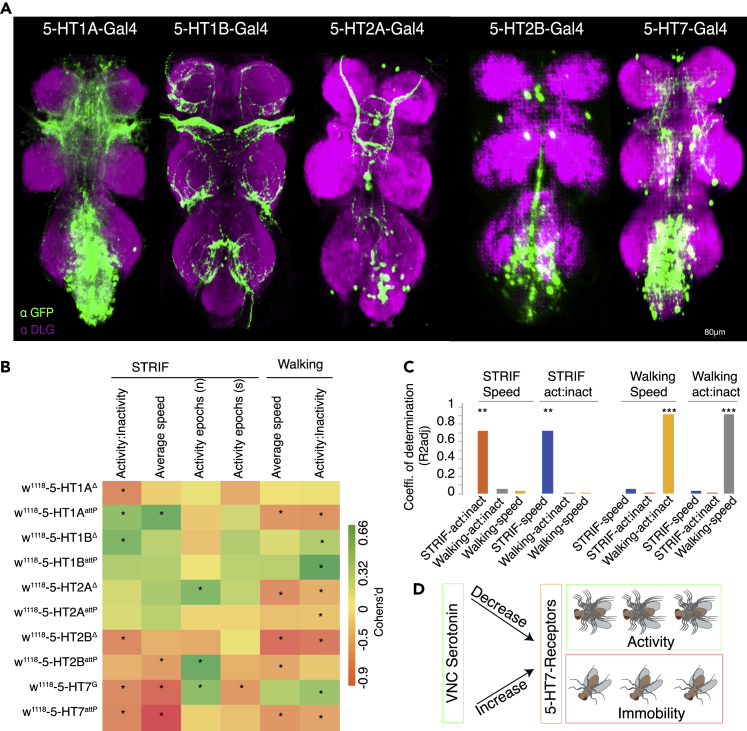
Serotonin uses 5-HT7 receptors during STRIF behavior (A) Maximum intensity projection of Gal4-driven expression of serotonin receptors in the VNC expressing mCD8:GFP and immunostained with neuropil antibodies (anti-dlg, magenta), scale bar: 80 μm. (B) Heatmap depicting changes in multiple parameters of 5-HT receptor mutants in STRIF and walking behavior. Plotted values represent Cohen’s *d* effect size for each STRIF and walking paradigms intervention. The values marked with asterisks represent statistically significant effect sizes between the control and experimental animals (p < 0.05). (C) Coefficient of determination (R-squared) for STRIF vs. walking parameters, the values marked with asterisks represent statistically significant effect size between the control and experimental animals (p < 0.05). Act:inact - activity-inactivity. (D) Model suggesting 5HT7 receptor-mediated serotonin-induced STRIF-immobility by having inhibitory effects on STRIF activity. Statistical analysis: Paired *t*-test. Two-sided permutation t-test-based p values are displayed as ∗ for p < 0.05, ∗∗ for p < 0.01, and ∗∗∗ for p < 0.001.

Similar to mammals, 5-HT receptors in *Drosophila* are G-protein-coupled-receptors (GPCRs)^37^ and, based on the mechanism of transduction, can be broadly classified into inhibitory (coupled to G_I_ proteins and inhibiting cAMP production), excitatory (coupled to G_s_ proteins and activating cAMP production), and PLCL-IP3 mediated excitatory (coupled to G_q_ proteins and increasing intracellular calcium).^38^ *Drosophila* expresses five 5-HT receptors, with at least one belonging to these three categories. Specifically, 5-HT1A and 5-HT1B receptors belong to the inhibitory receptor system, 5-HT7 receptors are excitatory, and 5-HT2A and 5-HT2B are from the PLC-IP3 transduction family. In a nutshell, 5-HT1A and 5-HT1B receptors inhibit serotonergic activity, and 5-HT2A, 5-HT2B, and 5-HT7 receptors enhance serotonergic activity. To compare the expression patterns of different serotonin receptors, we utilized recently published 5-HT receptor Gal4 drivers.^36^ Each receptor, Gal4, drives expression in the brain and the VNC, with some distinct neuropils (Figure 5A).

To study the effect of serotonin receptors on the fly STRIF response, we utilized two different mutant alleles of 5-HT receptors, which include recently published insertion alleles and deletion mutants.^27^^,^^36^^,^^39^^,^^40^^,^^41^^,^^42^ We studied their impact in heterozygous conditions because all 5-HT receptors mutants, except for 5-HT1B mutants, are lethal in homozygous states. We crossed these 5-HT receptor mutants with *w*^*1118*^ isogenic flies and compared the behavior of heterozygous F1 progeny with the behavior of *w*^*1118*^ isogenic flies. We observed variable results in STRIF response between deletion and insertion alleles except that of two 5-HT7 receptors mutant alleles. Both mutant alleles of 5-HT7 show reduced STRIF activity-inactivity ratios compared to *w*^*1118*^ isogenic flies (Figures 5B and S4A). We next studied speed changes, activity epoch numbers, and durations in the STRIF paradigm. Similar to activity-inactivity ratios, STRIF speed was consistently reduced in 5-HT7 mutant alleles (Figures 5B and S4B). The effects on other STRIF parameters, like activity epoch numbers and durations, were either inconsistent or had only a small effect across all mutant alleles (Figure 5B).

We next studied 5-HT receptor mutants in the walking paradigm and analyzed the activity-inactivity ratios of each receptor mutant. Interestingly, we observed very consistent elevated activity-inactivity ratios in both the heterozygous mutant alleles of 5-HT1B receptors and consistently reduced activity-inactivity ratios in 5-HT2A receptor mutants compared to *w*^*1118*^ isogenic flies Figures 5B and S4C); the effects on other 5-HT receptors were variable (Figures 5B and S4C). We next measured speed in the walking paradigm; out of all the mutant receptors, only 5-HT2B receptor mutant alleles show a reduced walking speed (Figures 5B and S4D). The effects obtained in the walking paradigm were different from the effects observed in the STRIF paradigm. We performed regression analysis between the effects obtained by 5-HT receptors alleles on STRIF and walking paradigm, and similar to our earlier observations, there is indeed a strong correlation between these two parameters (activity-inactivity ratios and speed) when analyzed within an assay; but these two parameters show no correlation on cross platforms (STRIF vs. walking, Figure 5C), which further corroborates our finding that locomotion-instructing serotonergic pathways are distinct from those STRIF-regulating serotonergic pathways. Overall, our data suggest that serotonin released in the VNC has a major effect on STRIF-activity via 5-HT7 receptors, distinct from its effect on walking (Figure 5D).

## Discussion

*Drosophila* uses distinct behavioral defensive strategies to combat threats or aversive cues, and these strategies are examples of fight-flight-freeze responses.^5^ Using several paradigms, various neuronal populations and pathways regulating the *Drosophila* defense responses have been identified.^5^^,^^43^^,^^44^ The state of captivity triggers stereotypical behaviors, to which animals resort for a limited period of time in attempts to escape. While physically fit animals may be capable of escaping from the trap conditions by wriggling, sustained immobility possibly represents a defensive strategy of feigning death, due to which predators may lose interest in pursuing the attack and loosen their grip, thereby increasing the chances of the prey to escape. However, how these behaviors are controlled and manifested in an animal is unclear. Here, we developed a simple paradigm with restrained flies and showed that (1) optogenetic activity and inactivity in a broader serotonin circuit enhance immobility and activity struggle, respectively, (2) serotonin cells in the VNC mediate immobility, and (3) the immobility states are mediated by 5-HT7 receptors located in the VNC. Below, we discuss these findings and present a preliminary model that summarizes the role of serotonin in modulating defensive states in restrained *Drosophila*.

### The STRIF response in restrained *Drosophila* is a defensive behavioral response

Alternating bursts of activity and immobility in restrained fruit flies may be associated with fleeing and freezing the animal, respectively,^7^^,^^13^^,^^45^ manifested in inescapable trap situations. Immobility is a defensive state, and it can make an animal less noticeable for detection by predators that react to movements. Therefore, immobility may represent an instinctive reaction and a protective strategy of prey. Here, we characterize the STRIF behavior in adult *Drosophila* flies, similar to that observed in vertebrates.^2^The behavioral feature of periods of activity and inactivity seen in the fly’s restrained state also resembles the behavior of rodents in the tail suspension test^46^ and forced swim test.^47^

### Multiple environmental and genetic factors influence the STRIF response

Female flies showed a higher STRIF-activity response than males. Our detection of sex differences in the STRIF assay may be consistent with the sex differences observed in other assays that measure behavioral outcomes after stress induction.^48^^,^^49^^,^^50^ These results also mirror the sex differences in humans diagnosed with major depression, generalized anxiety, and panic disorders.^51^^,^^52^ It is known that acute and chronic stress in rodents enhances and suppresses serotonin, respectively.^53^ Our finding that acute food deprivation in flies led to greater STRIF-immobility may be linked with enhanced activity of serotonergic neurons.^11^ Similarly, increased STRIF activity exhibited by sub-chronically immobilized and socially isolated flies may be associated with a suppressed serotonin system.^54^ Alternatively, these stressors could have altered the expression of postsynaptic serotonin receptors.

### Serotonergic modulation of the STRIF response

With repeated unsuccessful attempts to escape trap-like situations, behavior changes may manifest in abnormal repetitive responses caused by motivational frustration. Such responses may also indicate psychological changes^55^ that animals express through stereotypical behaviors. The inescapable conditions of captivity can also induce a progressive approach to immobility (motor inhibition), similar to learned helplessness, which may indicate the psychological suffering of the animal. Such a state may represent the animal’s higher-order mental processes, including human psychological states.^56^ In rodents, inescapable shock leads to a substantial increase in the release of serotonin from the raphe nuclei to other brain regions innervated by serotonergic neurons.^57^ Importantly, activation of serotonergic neurons has also been reported under other uncontrolled stressors, such as social defeat paradigms.^58^ Conversely, depletion of serotonin in the amygdala has been shown to reduce anxiety and fear responses in mice.^59^ We next explored how this seemingly simple behavior is regulated at the neuronal level in *Drosophila*. We utilized an advanced optogenetic toolkit and targeted serotonergic systems with multiple transgenic lines. We uncovered the role of serotonergic neurons in the VNC and receptors that promote immobility.

The effect of broader serotonin on the enhancement of immobility is generally consistent with Soubrie’s theory, which states that the main function of serotonin is to enhance behavioral inhibition.^60^ Activation and inactivation of serotonin neurons have previously been shown to reduce and enhance walking activity, respectively;^25^ however, in our STRIF paradigm, we observe that both optogenetic activation and inactivation of serotonergic cells mainly suppress STRIF average speed, while other parameters remain unaffected. Moreover, when we compared changes in parameters in 5-HT receptors in the walking and STRIF paradigm, we observed that while distinct receptors (5-HT1B and 5-HT2A) show differential effects on walking behavior, only 5-HT7 receptors affected STRIF-response. Although, when compared to previous observations made on the walking speed of some 5-HT receptor mutants,^25^ the results of our walking speed data are inconsistent. The inconsistency could be attributed to several differences between these studies, which include different receptor mutant alleles, genetic backgrounds (*CS* vs. *w*^*1118*^), and behavioral paradigms (startle vs. baseline). Overall, our data also suggest that serotonin mediates STRIF immobility, consistent with the idea that behavioral inhibition accurately describes serotonin function. We propose that several distinct neuronal pathways regulate serotonin-mediated higher-order functions in the VNC that are mediated by 5-HT7 receptors possibly situated on inhibitory neurons.

5-HT7 are excitatory receptors, and deletion of 5-HT7 receptors enhances fly STRIF-immobility, suggesting activity in certain VNC 5-HT neuronal clusters leads to increased activity, which could be mediated by these receptors that, when mutated, leads to an increase in immobility state (5-HT7). Alternatively, these receptors might be expressed on inhibitory neurons in the VNC and mediating STRIF-immobility. However more thorough analysis needs to be done to identify serotonin subclusters controlling activity and immobility in freely moving and restrained states. Our data suggest multiple serotonergic pathways in the VNC regulate fly immobility. 5-HT7 receptors have been implicated in various psychological disorders.^17^^,^^61^^,^^62^ Interestingly, 5-HT7 antagonists also exert antidepressant-like effects in the forced swim test.^63^ However, in our study, we observe that 5-HT7 receptor mutant flies show enhanced STRIF-immobility, which contradicts what has been reported earlier in mice.^63^

### Limitations of the study

Using *Drosophila*, a neurogenetic toolkit to actuate activity in serotonergic neurons and a novel escape paradigm, we have described here that changes in serotonergic neurocircuitry have a dichotomous influence on overall locomotor activity and escape response. However, there are a few limitations in this research. First, in the majority of the experiments, we have studied behavioral responses in males; however, given that sex differences have previously been identified in coping strategies^34^ and in response to neuroactive compounds^64^ in *Drosophila*, it will be interesting to study how female flies behave to serotonergic manipulations in the STRIF-based escape paradigm described in this study. Secondly, we have studied the role of serotonin at broader levels and identified the involvement of VNC in the regulation of STRIF response. However, the underlying circuitry controlling escape under restrained states could be much more complex, and smaller and unique subsets of serotonergic VNC neurons may control escape behavior distinctly in a network and isolation.^65^ Hence, driving neural actuators only in distinct serotonergic subsets and analyzing their effects on escape strategies, in combination with advanced techniques, such as calcium imaging in VNC, could shine light on the identity of individual circuits involved in escape behavior in restrained states and how they are distinct from cells driving locomotion. Thirdly, as mentioned earlier, STRIF behavior may be similar to the behavior displayed by rodents in the forced swim and tail suspension test paradigms. Genetic and neuronal manipulations of the serotonin system suggest that STRIF behavior could be used as a model for studying conserved mechanisms of depression or conducting screening for psychoactive drugs for potential antidepressants. Further studies are required to assess whether STRIF behavior has predictive value in establishing the effects of antidepressant compounds, which has been observed in other models of stress in *Drosophila*.^66^^,^^67^ Lastly, sustained activity and immobility may also have other behavioral and physiological correlations, including perturbed heart rate, respiration, defecation, and tremors. There are technical constraints measuring these parameters in both free and restrained flies; hence, we did not measure them. Further investigation will elucidate whether *Drosophila* exhibits these physiological changes and may reveal additional core conserved elements of this behavior with technical advancement. Nevertheless, 5-HT is known to alter heart rate in animals, including *Drosophila.*

## STAR★Methods

### Key resources table

**Table:** 

REAGENT or RESOURCE	SOURCE	IDENTIFIER
**Antibodies**		

Mouse anti-Dlg (1:50)	Developmental Studies Hybridoma Bank- DSHB	Cat# 4F3; RRID:AB_528203
Rat anti-5-HT (1:50)	Merck-Millipore	Cat#MAB-352; RRID: AB_11213564
Goat anti-Mouse 568 (1:200)	Thermo Fisher Scientific	Cat# A-11031; RRID: AB_144696
Rabbit anti-Rat 594 (1:200)	Thermo Fisher Scientific	Cat# A-21211; RRID: AB_10375432
Goat anti-Rat 488 (1:200)	Thermo Fisher Scientific	Cat # A-11006; RRID: AB_141373

**Experimental models: Organisms/strains**		

*Drosophila melanogaster:* Trh Gal4 (III)	Bloomington Stock Center (BDSC)	RRID:BDSC # 38389
*Drosophila melanogaster:* Tdc2 Gal4 (II)	BDSC	RRID:BDSC #9313
*Drosophila melanogaster:* R58E02-Gal4	BDSC	RRID:BDSC #41347
*Drosophila melanogaster:* Ser-T- Gal4 (III)	BDSC	RRID:VDRC #VT021853
*Drosophila melanogaster:* 5-HT1A-Gal4 (II)	BDSC	RRID:BDSC #84588
*Drosophila melanogaster*: 5-HT1B-Gal4 (II)	BDSC	RRID:BDSC #86276
*Drosophila melanogaster:* 5-HT2A-Gal4	BDSC	RRID:BDSC #86277
*Drosophila melanogaster;* 5-HT2B-Gal4	BDSC	RRID:BDSC #84445
*Drosophila melanogaster*: 5-HT7-Gal4(III)	BDSC	RRID:BDSC #86279
*Drosophila melanogaster*: UAS-Chrimson (X)	BDSC	RRID:BDSC #55134
*Drosophila melanogaster:* UAS-GtACR1 (III)	N/A	N/A
*Drosophila* melanogaster*:* 20X-UAS-hexameric 6X GFP	BDSC	RRID:BDSC #52262
*Drosophila melanogaster:* 20X-UAS-mcD8-GFP	BDSC	RRID:BDSC#32185
*Drosophila melanogaster:* Tsh-Gal80; MKRS/Tb	Gift from Julie Simpson	N/A
*Drosophila melanogaster:* Tsh-Gal80; UAS- Chrimson/Tb	This Study	N/A
*Drosophila melanogaster:* Trh-RNAi1 (A + B) RNAi	Gift from Julie Simpson	N/A
*Drosophila melanogaster:* Tub < Gal80>; Tsh- LexA, LexA-OP, Flp; Chrimson/Tb	Gift from Richard S Mann	N/A
*Drosophila melanogaster*: 5-HT1A^[attp].^ (II)	BDSC	RRID:BDSC #84704
*Drosophila melanogaster*: 5-HT1B^[attp]^ (II)	BDSC	RRID:BDSC #84443
*Drosophila melanogaster:* 5-HT2A^[attp].^ (III)	BDSC	RRID:BDSC #84444
*Drosophila melanogaster*: 5-HT2B^[attp]^ (III)	BDSC	RRID:BDSC #84445
*Drosophila melanogaster*: 5-HT7^[attp]^ (III)	BDSC	RRID:BDSC #84446
*Drosophila melanogaster*: 5-HT1A[Delta5kb] (II)	BDSC	RRID:BDSC #27640
*Drosophila melanogaster*: 5-HT1B[DeltaIII-V] (II)	BDSC	RRID:BDSC #55846
*Drosophila melanogaster:* 5-HT2A[c1644] (III)	BDSC	RRID:BDSC #4830
*Drosophila melanogaster:* 5-HT2B[MI05208] (III)	BDSC	RRID:BDSC #42994
*Drosophila melanogaster*: 5-HT7[Gal4] (III)	BDSC	RRID:BDSC #86279
*Drosophila melanogaster*: w1118 [iso]	BDSC	RRID:BDSC #5905
*Drosophila melanogaster*: Canton-S	BDSC	RRID:BDSC #64349
*Drosophila melanogaster*: Oregon-R-C	BDSC	RRID:BDSC #5

### Resource availability

#### Lead contact

Further information and requests for resources and reagents should be directed to and fulfilled by the lead contact, Dr. Mohammad Farhan (mohammadfarhan@hbku.edu.qa.)

#### Materials availability

All the fly lines generated in this study are available upon request from the lead contact without restrictions.

### Experimental model and subject details

#### *Drosophila* strains and fly medium

All fly strains were kept in incubators at a temperature of 25°C under 12h:12h light-dark cycles and 70% humidity in Darwin Chambers (IN084-AA-LT-DA-MP). F1 offspring derived from the driver and responder lines crossed with w1118 isogenic flies (Bloomington Drosophila Stock Center (BDSC), # 5905) were used as genotypic controls. All fly strains used for the experiments were reared on Nutri-Fly MF food medium (Genesee Scientific, Cat #66–116). In short, for 1 L of food, 177 g of NutriFly medium powder was dissolved in 1 L of water and boiled. The food was cooled down to 70–75°C. Right after, food preservatives and antifungal chemicals, such as 4.9 mL of Propionic acid (Merck-Sigma, Cat #8006052500) and 10 mL of 10% Nipagene (Tegosept, Genesee Scientific, Cat #20–259) prepared in absolute ethanol, were added to the molten food medium and mixed. The prepared medium was immediately poured into plastic vials and bottles.

### Method details

#### Fly rearing for behavioral experiments

All flies used for behavioral experiments were grown at either 25°C or 18°C in an incubator (PHCbi, MIR254). Genetic crosses used for behavioral experiments were kept in an incubator at 25°C under 12h:12h light-dark cycles. For optogenetic experiments, male flies of 2–3 days of age were collected by anesthesia on ice. The flies were then transferred to the All-Trans-Retinal (ATR, Carbosynth, #16-31-4) food medium (1 mM) for 2–3 days at 25°C in the dark. ATR was dissolved in 95% EtOH. Crosses were grown in dark wrapped with aluminum foil to avoid any effects by optogenetic activation during their developmental stages.

##### STruggle Response in Immobilized Fly (STRIF) assay

The STruggle Response in Immobilized Fly (STRIF) assay was developed in the laboratory to record the struggle behavior of restrained flies. Male flies of 4–5 days of age were anesthetized on ice for 5–10 s. The flies were glued onto a glass slide with a transparent odorless glue (Deli StickUp 502 Super Glue No. 7146) on their thorax and left alone to rest for 10–15 min. The motor activity of these restrained flies was recorded for 5 min using a camera (Leica IC90E) and LAX3.0 software (Leica) at a speed of 30 frames per second.

##### Walking assay

Fly locomotion was assayed using the *Drosophila* Arousal Threshold (DART) system.^24^ Briefly, the flies were transferred to 15 mm arenas, and their activity was recorded for 5 min at a speed of 15 frames per second using a webcam equipped with a wide-angle lens. MATLAB scripts were used to track the fly location.^24^ The activity and inactivity time ratios, as well as the average speed, were calculated for each fly.

##### Optogenetics

For optogenetic activation experiments, restrained flies carrying Chrimson^68^ were illuminated with peak red light (intensity-RGB-255,0,0) from a PC-controlled LED mini-projector (OPTOMA, ML750). For optogenetic inactivation experiments, flies carrying GtACR1^35^ were illuminated with peak green light (Higher intensity, RGB-0.255,0; lower intensity, RGB-0.127,0) from an LED micro-projector. The LED mini-projector was placed near (∼14 cm) to the behavioral setup.

##### Light intensity measurements

The light intensity of the projector illumination was measured for the red and green wavelengths (λ 635 and 532), which were used in the optogenetics experiments. The light power was measured in a dark room by collecting light photons on the sensor area of 1 cm^2^. An optical power meter (Thorlabs PM400) connected to an integrated optical sensor (Thorlabs S120C) was used for the light power measurements. The power meter was reset to zero before each set of measurements and placed in the same place where fly recordings were performed.

##### Video acquisition

Videos of the restrained flies were shot in RGB format at a speed of 30 frames per second from a camera mounted with a Leica microscope. Image acquisition software (LAX3.0, Leica) was used to record the activity of restrained flies. Metadata characterizing the genotype of fly according to its location on the experimental video were recorded for each experiment.

##### Fly detection and movement tracking

To track the movements of the restrained flies, we utilized a custom-made script written using OpenCV2.0^69^^,^^70^ and Python 3.0. The following libraries from Python 3.0 were used: numpy, scipy, pandas, scikit-learn matplotlib, seaborn, opencv, scikit-image pillow, and spyder. Briefly, in the captured video, flies were detected as blobs using Otsu thresholding, followed by morphological operations for cleaning, connected components analysis, and size thresholding^71^^,^^72^ for determining the blob. For each fly, the region of interest (ROI) was defined as a square around the detected fly blob centroid with a width corresponding to the maximum width/height of the detected fly. Thus, the entire body of each fly was captured in the ROI. Motion activity was measured as the absolute difference between consecutive video frames. Any image interference or noise (salt and pepper) was removed using the median filter and minimum difference filters^73^ in OpenCV2.0. For a given ROI, STRIF-speed was calculated as the average of pixel frame differences between alternating frames. Background activity outside the ROI was also calculated and used for video flash detection. The background of the entire analyzed image was fixed. Multiple videos were analyzed using a group analysis function, and metadata information was used to assign the genotype of each fly. In optogenetic experiments, the light-on (flash-on) time was detected in the videos as the change in RGB values. The activity of each fly and the flash-on time were exported to a CSV file.

##### Behavioral metrics and data analysis

When restrained, a fly exhibits several behavioral reactions, including epochs of enhanced activity and inactivity, grooming episodes, flapping of wings, abdominal thrusts, and defecation. We measured collective behavior of restrained flies using our custom-built python script, we detected various behavioral parameters: the average total time spent in activity and inactivity, the number of activity and inactivity epochs, the average time spent in an active or inactive epoch, as well as the frequency of activity and inactivity. To classify behavior into activity and inactivity, we used a 5 second threshold: a fly inactive for five or more seconds was considered immobile. For non-optogenetic experiments, the ratio of time spent in activity vs. inactivity was used as the primary matrix for studying the STRIF response. For optogenetic experiments, the time spent in activity before vs. after optogenetic illumination was compared pairwise for each fly. The total time spent in activity and inactivity was named STRIF-activity and STRIF-inactivity, respectively.

##### Immunohistochemistry and confocal microscopy

The fly’s brain and VNC were dissected in isotonic PBS (PBS) and fixed in 4% Paraformaldehyde (PFA) for 20 min at room temperature. Two quick washes were performed with PBST (PBS with 1% Triton X-100) to completely remove the fixative (PFA), followed by four washes with PBST wash buffer for 1 h at 15 min intervals. The brain and VNC samples were then blocked for 30 min using a blocking solution (PBST with a 1% BSA) and then incubated with primary antibodies overnight at 4°C on a rotator at 50 rpm. After completion of incubation with primary antibodies, the samples were washed with PBST four times at 15 min intervals. The samples were then incubated with secondary antibodies for 3–4 h at room temperature. Samples were washed with PBST four times at 15 min intervals. The processed samples were then mounted on a glass slide using the Vectashield mounting medium (Vector Laboratories). Confocal images were obtained using a Nikon A1 confocal microscope. Images were analyzed in ImageJ and presented as Maximum Intensity Projection (MIP).

### Quantification and statistical analysis

The error bars in all data figures represent 95% confidence intervals (95CI) for the mean difference. All data points are presented as means unless otherwise specified. For comparisons between two independent groups, the CIs of primary data and contrasts were calculated using the bias-corrected and accelerated bootstrapping (BCa) method (bootci function). The bootstrap method of calculating effect size distribution is robust for non-normal data, especially at large sample sizes. The p values were computed using the permutation t-test. This statistical testing was combined with the calculation of the effect sizes, we calculated and presented two standardized effect sizes. The standardized effect sizes were measured as Glass delta (Δ) and Cohen’s *d*. Cohen’s *d* or other standardized effects do not get influenced by the study design and remain stable across different versions of the same measurements^74^ (for example activity-inactivity ratios in optogenetic vs non-optogenetic, STRIF vs walking etc.). Hence, using Cohen’s *d*, we compared the effect sizes across the manipulations in STRIF and walking assays. Glass delta (Δ) was calculated by dividing the mean difference (experiment - controls) by the SD of controls and Cohen’s *d* was calculated by dividing the mean difference (experiment - controls) by the pooled SD of controls and experiments.^75^ Glass delta (Δ) and Cohen’s *d* indicates how different the two groups are from each other. For example, Δ or *d* equal to 1 show that the two groups differ by one SD. In accordance with standard practice, the magnitude of the effect is interpreted as ‘negligible’ (Δ, *d* < 0.2), ‘small’ (0.2 < Δ or *d* < 0.5), ‘moderate’ (0.5 < Δ or *d* < 0.8), or ‘large’ (Δ or *d* > 0.8).^76^ STRIF sample sizes (typically n *=* 45) had a power of >0.8 at α = 0.05 and *d* = 0.6. Linear regression was performed in MATLAB (LinearModel.fit function).

## Data Availability

All raw data and python scripts will be provided on request. Further information on code availability to request lead contact (mohammadfarhan@hbku.edu.qa) Microscopy data reported in this data will be provided upon request to lead contact. Any additional information required to reanalyze the data reported in this paper is available from the lead contact upon request.

## Supplemental information

Document S1. Figures S1–S4

Table S1. Statistical Data of STRIF Parameters-optogenetics, related to Figures 2–4

Table S2. Statistical Data of Walking parameters -optogenetics, related to Figures 2 and 3

Table S3. Statistical Data of STRIF Parameters-non-optogenetics, related to Figures 1, 3, S1, S3, and S4

Table S4. Statistical Data of Walking parameters -non-optogenetics, related to Figures 1, 3, S2, and S4

Table S5. Genotypes used in this study, related to STAR Methods

## References

[1] Fanselow M.S., Hoffman A.N., Zhuravka I. (2019). Timing and the transition between modes in the defensive behavior system. Behav. Processes.

[2] Seo C., Guru A., Jin M., Ito B., Sleezer B.J., Ho Y.Y., Wang E., Boada C., Krupa N.A., Kullakanda D.S. (2019). Intense threat switches dorsal raphe serotonin neurons to a paradoxical operational mode. Science.

[3] Gallup G.G., Rager D.R. (1996). Motor Activity and Movement Disorders.

[4] Perrins R., Walford A., Roberts A. (2002). Sensory activation and role of inhibitory reticulospinal neurons that stop swimming in hatchling frog tadpoles. J. Neurosci..

[5] Gibson W.T., Gonzalez C.R., Fernandez C., Ramasamy L., Tabachnik T., Du R.R., Felsen P.D., Maire M.R., Perona P., Anderson D.J. (2015). Behavioral responses to a repetitive visual threat stimulus express a persistent state of defensive arousal in drosophila. Curr. Biol..

[6] Liang F., Xiong X.R., Zingg B., Ji X.y., Zhang L.I., Tao H.W. (2015). Sensory cortical control of a visually induced arrest behavior via corticotectal projections. Neuron.

[7] Zacarias R., Namiki S., Card G.M., Vasconcelos M.L., Moita M.A. (2018). Speed dependent descending control of freezing behavior in Drosophila melanogaster. Nat. Commun..

[8] McNaughton N., Corr P.J. (2004). A two-dimensional neuropsychology of defense: fear/anxiety and defensive distance. Neurosci. Biobehav. Rev..

[9] Card G.M. (2012). Escape behaviors in insects. Curr. Opin. Neurobiol..

[10] Mobbs D., Hagan C.C., Dalgleish T., Silston B., Prévost C. (2015). The ecology of human fear: survival optimization and the nervous system. Front. Neurosci..

[11] Evans D.A., Stempel A.V., Vale R., Branco T. (2019). Cognitive control of escape behaviour. Trends Cogn. Sci..

[12] Gallup G.G. (1977). Tonic immobility: the role of fear and predation. Psychol. Rec..

[13] Fanselow M.S. (1994). Neural organization of the defensive behavior system responsible for fear. Psychon. Bull. Rev..

[14] Kozlowska K., Walker P., McLean L., Carrive P. (2015). Fear and the defense cascade: clinical implications and management. Harv. Rev. Psychiatr..

[15] Kalaf J., Vilete L.M.P., Volchan E., Fiszman A., Coutinho E.S.F., Andreoli S.B., Quintana M.I., De Jesus Mari J., Figueira I. (2015). Peritraumatic tonic immobility in a large representative sample of the general population: association with posttraumatic stress disorder and female gender. Compr. Psychiatr..

[16] Volchan E., Rocha-Rego V., Bastos A.F., Oliveira J.M., Franklin C., Gleiser S., Berger W., Souza G.G.L., Oliveira L., David I.A. (2017). Immobility reactions under threat: a contribution to human defensive cascade and PTSD. Neurosci. Biobehav. Rev..

[17] Mohammad F., Aryal S., Ho J., Stewart J.C., Norman N.A., Tan T.L., Eisaka A., Claridge-Chang A. (2016). Ancient anxiety pathways influence Drosophila defense behaviors. Curr. Biol..

[18] Ogawa S.K., Cohen J.Y., Hwang D., Uchida N., Watabe-Uchida M. (2014). Organization of monosynaptic inputs to the serotonin and dopamine neuromodulatory systems. Cell Rep..

[19] Bargmann C.I. (2012). Beyond the connectome: how neuromodulators shape neural circuits. Bioessays.

[20] McBride R.L., Klemm W.R. (1969). Mechanisms of the immobility reflex ('animal hypnosis’) 1. Influences of repetition of induction, restriction of auditory-visual input and destruction of brain areas. Commun. Behav. Biol. (Ser A).

[21] Klemm W.R. (1976). Identity of sensory and motor systems that are critical to the immobility reflex (“animal hypnosis”). J. Neurosci. Res..

[22] Sitaraman D., Kramer E.F., Kahsai L., Ostrowski D., Zars T. (2017). Discrete serotonin systems mediate memory enhancement and escape latencies after unpredicted aversive experience in Drosophila place memory. Front. Syst. Neurosci..

[23] Barker A.J., Baier H. (2015). Sensorimotor decision making in the Zebrafish tectum. Curr. Biol..

[24] Faville R., Kottler B., Goodhill G.J., Shaw P.J., Van Swinderen B. (2015). How deeply does your mutant sleep? Probing arousal to better understand sleep defects in Drosophila. Sci. Rep..

[25] Howard C.E., Chen C.L., Tabachnik T., Hormigo R., Ramdya P., Mann R.S. (2019). Serotonergic modulation of walking in Drosophila. Curr. Biol..

[26] Yuan Q., Lin F., Zheng X., Sehgal A. (2005). Serotonin modulates circadian entrainment in Drosophila. Neuron.

[27] Yuan Q., Joiner W.J., Sehgal A. (2006). A sleep-promoting role for the Drosophila serotonin receptor 1A. Curr. Biol..

[28] Dierick H.A., Greenspan R.J. (2007). Serotonin and neuropeptide F have opposite modulatory effects on fly aggression. Nat. Genet..

[29] Yildizoglu T., Weislogel J.M., Mohammad F., Chan E.S.Y., Assam P.N., Claridge-Chang A. (2015). Estimating information processing in a memory system: the utility of meta-analytic methods for genetics. PLoS Genet..

[30] Graeff F.G., Zangrossi H. (2010). The dual role of serotonin in defense and the mode of action of antidepressants on generalized anxiety and panic disorders. Cent. Nerv. Syst. Agents Med. Chem..

[31] Waider J., Popp S., Lange M.D., Kern R., Kolter J.F., Kobler J., Donner N.C., Lowe K.R., Malzbender J.H., Brazell C.J. (2017). Genetically driven brain serotonin deficiency facilitates panic-like escape behavior in mice. Transl. Psychiatry.

[32] Klapoetke N.C., Murata Y., Kim S.S., Pulver S.R., Birdsey-Benson A., Cho Y.K., Morimoto T.K., Chuong A.S., Carpenter E.J., Tian Z. (2014). Independent optical excitation of distinct neural populations. Nat. Methods.

[33] Alekseyenko O.V., Lee C., Kravitz E.A. (2010). Targeted manipulation of serotonergic neurotransmission affects the escalation of aggression in adult male drosophila melanogaster. PLoS One.

[34] Videlier M., Rundle H.D., Careau V. (2019). Sex-specific among-individual covariation in locomotor activity and resting metabolic rate in Drosophila melanogaster. Am. Nat..

[35] Mohammad F., Stewart J.C., Ott S., Chlebikova K., Chua J.Y., Koh T.W., Ho J., Claridge-Chang A. (2017). Optogenetic inhibition of behavior with anion channelrhodopsins. Nat. Methods.

[36] Deng B., Li Q., Liu X., Cao Y., Li B., Qian Y., Xu R., Mao R., Zhou E., Zhang W. (2019). Chemoconnectomics: mapping chemical transmission in Drosophila. Neuron.

[37] Saudou F., Hen R. (1994). 5-Hydroxytryptamine receptor subtypes in vertebrates and invertebrates. Neurochem. Int..

[38] Nichols D.E., Nichols C.D. (2008). Serotonin receptors. Chem. Rev..

[39] Gasque G., Conway S., Huang J., Rao Y., Vosshall L.B. (2013). Small molecule drug screening in Drosophila identifies the 5HT2A receptor as a feeding modulation target. Sci. Rep..

[40] Schaerlinger B., Launay J.M., Vonesch J.L., Maroteaux L. (2007). Gain of affinity point mutation in the serotonin receptor gene 5-HT 2Dro accelerates germband extension movements during Drosophila gastrulation. Dev. Dyn..

[41] Nagarkar-Jaiswal S., Lee P.T., Campbell M.E., Chen K., Anguiano-Zarate S., Gutierrez M.C., Busby T., Lin W.W., He Y., Schulze K.L. (2015). A library of MiMICs allows tagging of genes and reversible, spatial and temporal knockdown of proteins in Drosophila. Elife.

[42] Qian Y., Cao Y., Deng B., Yang G., Li J., Xu R., Zhang D., Huang J., Rao Y. (2017). Sleep homeostasis regulated by 5HT2b receptor in a small subset of neurons in the dorsal fan-shaped body of drosophila. Elife.

[43] Li J., Zhang W., Guo Z., Wu S., Jan L.Y., Jan Y.N. (2016). A defensive kicking behavior in response to mechanical stimuli mediated by Drosophila wing margin bristles. J. Neurosci..

[44] Liu C., Zhang B., Zhang L., Yang T., Zhang Z., Gao Z., Zhang W. (2020). A neural circuit encoding mating states tunes defensive behavior in Drosophila. Nat. Commun..

[45] Eilam D. (2005). Die hard: a blend of freezing and fleeing as a dynamic defense - implications for the control of defensive behavior. Neurosci. Biobehav. Rev..

[46] Steru L., Chermat R., Thierry B., Simon P. (1985). The tail suspension test: a new method for screening antidepressants in mice. Psychopharmacology (Berl).

[47] Porsolt R.D., Bertin A., Jalfre M. (1978). “Behavioural despair” in rats and mice: strain differences and the effects of imipramine. Eur. J. Pharmacol..

[48] Neckameyer W.S., Nieto-Romero A.R. (2015). Response to stress in Drosophila is mediated by gender, age and stress paradigm. Stress.

[49] Batsching S., Wolf R., Heisenberg M. (2016). Inescapable stress changes walking behavior in flies - learned helplessness revisited. PLoS One.

[50] Ries A.S., Hermanns T., Poeck B., Strauss R. (2017). Serotonin modulates a depression-like state in Drosophila responsive to lithium treatment. Nat. Commun..

[51] Marks I.M. (1987).

[52] Weissman M.M., Olfson M. (1995). Depression in women: implications for health care research. Science.

[53] Mahar I., Bambico F.R., Mechawar N., Nobrega J.N. (2014). Stress, serotonin, and hippocampal neurogenesis in relation to depression and antidepressant effects. Neurosci. Biobehav. Rev..

[54] Ahmad A., Rasheed N., Banu N., Palit G. (2010). Alterations in monoamine levels and oxidative systems in frontal cortex, striatum, and hippocampus of the rat brain during chronic unpredictable stress. Stress.

[55] Clubb R., Mason G.J. (2007). Natural behavioural biology as a risk factor in carnivore welfare: how analysing species differences could help zoos improve enclosures. Appl. Anim. Behav. Sci..

[56] Garner J.P., Mason G.J. (2002). Evidence for a relationship between cage stereotypies and behavioural disinhibition in laboratory rodents. Behav. Brain Res..

[57] Grahn R.E., Will M.J., Hammack S.E., Maswood S., McQueen M.B., Watkins L.R., Maier S.F. (1999). Activation of serotonin-immunoreactive cells in the dorsal raphe nucleus in rats exposed to an uncontrollable stressor. Brain Res..

[58] Amat J., Aleksejev R.M., Paul E., Watkins L.R., Maier S.F. (2010). Behavioral control over shock blocks behavioral and neurochemical effects of later social defeat. Neuroscience.

[59] Johnson P.L., Molosh A., Fitz S.D., Arendt D., Deehan G.A., Federici L.M., Bernabe C., Engleman E.A., Rodd Z.A., Lowry C.A., Shekhar A. (2015). Pharmacological depletion of serotonin in the basolateral amygdala complex reduces anxiety and disrupts fear conditioning. Pharmacol. Biochem. Behav..

[60] Soubrié P. (1986). Reconciling the role of central serotonin neurons in human and animal behavior. Behav. Brain Sci..

[61] de Paula B.B., Leite-Panissi C.R.A. (2016). Distinct effect of 5-HT1A and 5-HT2A receptors in the medial nucleus of the amygdala on tonic immobility behavior. Brain Res..

[62] Hedlund P.B., Huitron-Resendiz S., Henriksen S.J., Sutcliffe J.G. (2005). 5-HT7 receptor inhibition and inactivation induce antidepressantlike behavior and sleep pattern. Biol. Psychiatr..

[63] Yohn C.N., Gergues M.M., Samuels B.A. (2017). The role of 5-HT receptors in depression Tim Bliss. Mol. Brain.

[64] Sharma A., Mohammad F., Singh P. (2009). Gender differences in a drosophila transcriptomic model of chronic pentylenetetrazole induced behavioral deficit. PLoS One.

[65] Albin S.D., Kaun K.R., Knapp J.M., Chung P., Heberlein U., Simpson J.H. (2015). A subset of serotonergic neurons evokes hunger in adult Drosophila. Curr. Biol..

[66] Araujo S.M., Poetini M.R., Bortolotto V.C., de Freitas Couto S., Pinheiro F.C., Meichtry L.B., de Almeida F.P., Santos Musachio E.A., de Paula M.T., Prigol M. (2018). Chronic unpredictable mild stress-induced depressive-like behavior and dysregulation of brain levels of biogenic amines in Drosophila melanogaster. Behav. Brain Res..

[67] Hibicke M., Nichols C.D. (2022). Validation of the forced swim test in Drosophila, and its use to demonstrate psilocybin has long-lasting antidepressant-like effects in flies. Sci. Rep..

[68] Klapoetke N.C., Murata Y., Kim S.S., Pulver S.R., Birdsey-Benson A., Cho Y.K., Morimoto T.K., Chuong A.S., Carpenter E.J., Tian Z. (2014). Addendum: independent optical excitation of distinct neural populations. Nat. Methods.

[69] Gary Rost B., Adrian K. (2008).

[70] Gary B., Kaehler A. (2012).

[71] Otsu N. (1979). Threshold selection method from gray-level histograms. IEEE Trans. Syst. Man Cybern..

[72] Huang D.Y., Wang C.H. (2009). Optimal multi-level thresholding using a two-stage Otsu optimization approach. Pattern Recogn. Lett..

[73] Huang T., Yang G., Tang G. (1979). A fast two-dimensional median filtering algorithm. IEEE Trans. Acoust. Speech Signal Process..

[74] Baguley T. (2009). Standardized or simple effect size: what should be reported?. Br. J. Psychol..

[75] Claridge-Chang A., Assam P.N. (2016). Estimation statistics should replace significance testing. Nat. Methods.

[76] Cumming G. (2012).

