# Supplementary material for: Serotonin distinctly controls behavioral states in restrained and freely moving Drosophila
Source: iScience. 2022 Dec 28;26(1):105886. doi: 10.1016/j.isci.2022.105886 (PMC9840979; doi:10.1016/j.isci.2022.105886)
Supplement: Document S1. Figures S1–S4 [file mmc1.pdf]

iScience, Volume *26*

Supplemental information

Serotonin distinctly controls behavioral states in restrained and freely moving *Drosophila*

Swetha B.M. Gowda, Ayesha Banu, Safa Salim, Kadir A. Peker, and Farhan Mohammad

Supplemental Information

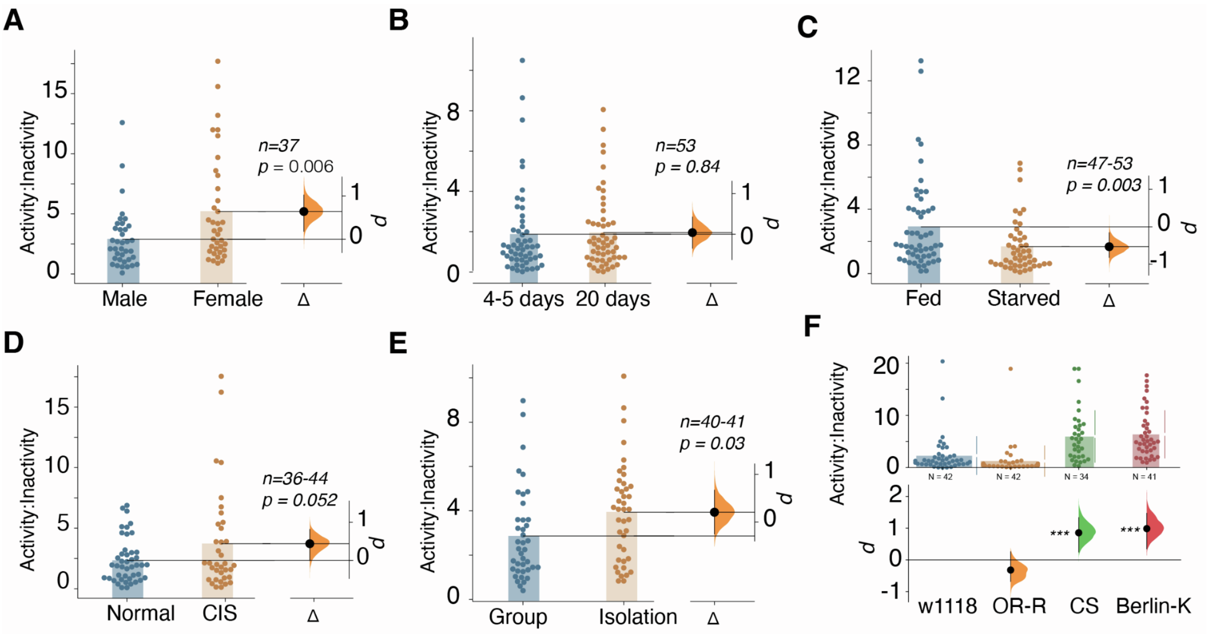

Figure S1. Potential factors influencing STRIF behavior, Related to Figure 1.

A. Activity-inactivity ratio showing that females exhibited higher levels of STRIF-activity compared to males (*n* = 37, Δ =1.05, *d* = 0.7 [95CI 0.24, 1.1], *p = 0.006)*. **B**. There was no significant difference in STRIF-activity in young vs. older flies (*n* = 53, Δ = 0.04, *d* = 0.04, [95CI -0.34, 0.43], *p = 0.83)*. **C**. Starved flies displayed decreased STRIF response compared to fed flies (*n* = 47-53, Δ =0.50, *d* = -0.58, [95CI -0.92, -0.077], *p = 0.003)*. **D**. Chronical immobilization stress (CIS) flies showed slightly increased STRIF-activity in comparison to freely moving flies (*n* = 36-44, Δ = 0.72, *d* = 0.42, [95CI -0.031, 0.79], *p = 0.052*). **E**. Socially isolated flies showed increased STRIF-activity compared to grouped flies. Each dot on the scatter plot represents the STRIF response of one fly (*n* = 40-41, Δ = 0.47, *d* = 0.47, [95CI 0.006, 0.96], *p = 0.03*). **F**. Activity-inactivity ratio of different genotypes, indicating that *CS* and *Berlin-K* flies showed more STRIF-activity compared to OR and w^{1118} flies; w^{1118} was used as a shared control (*n* = 34-42, w^{1118} *-OR-R*, Δ = -0.28, *d* = -0.31, [95CI -0.68, 0.259], *p = 0.17; w^{1118} -CS*, Δ = 1.0, *d* = 0.85, [95CI 0.248, 1.33], *p = 0.0006; w^{1118} Berlin-K*, Δ = 1.11, *d* = 0.98, [95CI 0.34, 1.47], *p = 0.0001*). The experiment-control differences are displayed as the effect size (Cohen's *d*) with an error curve and 95CI. Permutation t-test *P* values (two-tailed) were used for testing significance.

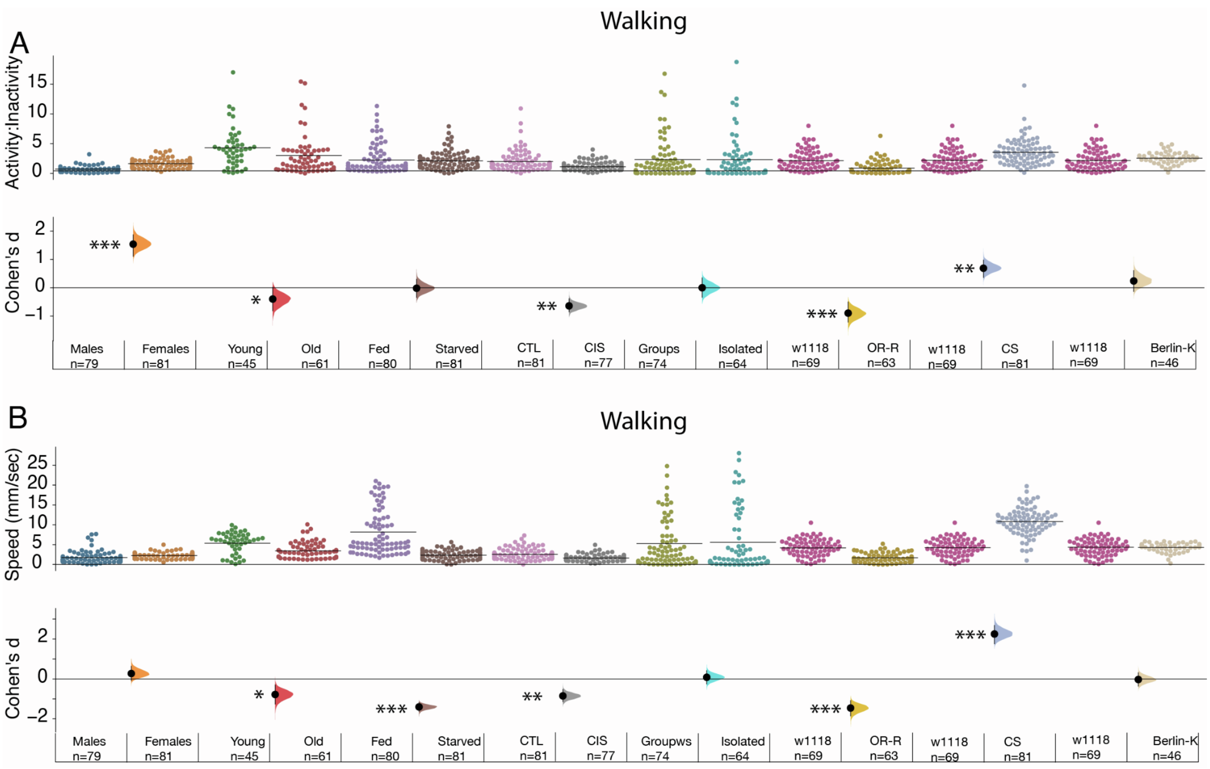

Figure S2. Potential factors influencing walking behaviors in freely moving flies, Related to Figure 1.

A. Activity-inactivity ratio of eight factors-gender (n = 79-81, Δ = 2.06, *d* = 1.5, [95CI 1.1, 1.9], *p = 0.0001)*, age (n = 45-61, Δ = -0.39,*d* = -0.4, [95CI -0.81, 0.02], *p = 0.05)*, satiety (n = 80-81, Δ =-0.0, *d* = -0.02, [95CI -0.34, 0.3], *p = 0.93)*, chronic immobility (n = 77-81, Δ =-0.5, *d* = -0.64, [95CI -0.85, -0.39], *p = 0.001)*, isolation (n = 64-74, Δ = 0.16 *d* = 0.003, [95CI -0.33, 0.4], *p = 0.99)*, and the genotypes w^{1118}, OR-R, CS, and Berlin-K (OR-R, n = 63-69, Δ = -0.76,*d* = -0.89, [95CI -1.2, -0.49], *p = 0.001;* CS, n = 69-81, Δ = 0.8, *d* = 0.69, [95CI 0.38, 0.98], *p = 0.0001;* Berlin-K, n = 46-69, Δ = 0.21, *d* = 0.24, [95CI -0.13, 0.61], *p = 0.21)*. **B.** Walking speed of eight factors- gender (n = 79-80, Δ = 0.21, *d* = 0.27, [95CI -0.08, 0.64], *p = 0.093)*, age (n = 45-60, Δ = -0.7, *d* = -0.78, [95CI -1.3, -0.3], *p = 0.005)*, satiety (n = 80-81, Δ = -1.01, *d* = -1.4, [95CI -1.6, -1.2], *p = 0.0001)*, chronic immobility (n = 77-81, Δ = -0.7, *d* = -0.85, [95CI -1.1, -0.53], *p = 0.0001)*, isolation (n = 65-74, Δ =0.01, *d* = 0.08, [95CI -025, 0.42], *p = 0.64)*, and the genotypes w^{1118}, OR-R, CS, and Berlin-K (OR-R, n = 63-69, Δ = -1.3, *d* = -1.5, [95CI -1.9, 1.1], *p = 0.0001;* CS, n=69-81, Δ = 3.08, *d* = 2.3, [95CI 1.8, 2.7], *p = 0.0001;* Berlin-K, n = 46-69, Δ =-002, *d* = -0.03, [95CI -0.4, 0.31], *p = 0.87)*.

Movie S1-S9. Restrained fly exhibiting activity, immobility, abdomen thrust and wing movements and wing flapping behaviors, **Related to Figure 1.**

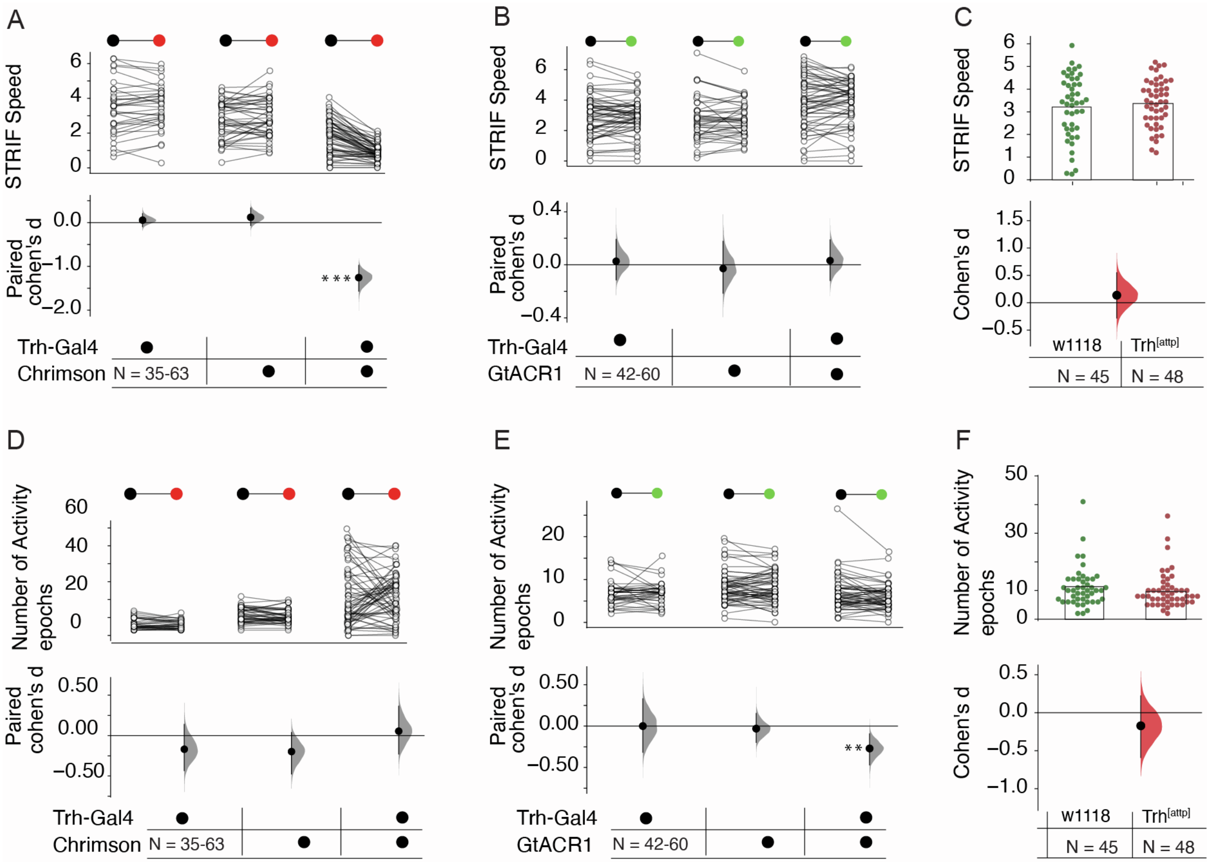

Figure S3. The effect of serotonin activation and inactivation on STRIF speed and the number of activity epochs., Related to Figure 2 and 3.

A. Optogenetic activity in the broader serotonin system (*Trh-Gal4-UAS-Chrimson)* reduced STRIF speed (n = 65, Δ = -1.09, *d* = -1.31, [95CI -1.65, -1.0], *p = 0.0001*). **B**. Optogenetic silencing of the broader serotonin system (*Trh-Gal4-UAS-GtACR1)* did not affect STRIF speed (n = 60, Δ = 0.03, *d* = 0.03, [95CI 0.13, 1.02], *p = 0.008*). **C**. *Trh*$^{[attpP]}$ homozygous mutant flies were comparable to *w*^{1118} isogenic flies (n = 48, Δ = 0.12, *d* = 0.13, [95. CI -0.28, 0.54], *p = 0.51*). **D**. Optogenetic activity in the broader serotonin system did not affect the number of activity epochs (n = 63, Δ = 0.18, *d* = 0.18, [95CI -0.16, 0.57], *p = 0.3*). **E**. Optogenetic inactivity in the broader serotonin system reduced the number of activity epochs (n = 60, Δ = 0.27, *d* = -0.27, [95.0%CI -0.47, -0.09], *p = 0.008*)**. F.** *Trh*$^{[attpP]}$ homozygous mutant flies were comparable to *w*^{1118} flies (n = 48, Δ = -0.21, *d* = -0.24, [95CI -0.659, 0.175], *p = 0.262*).

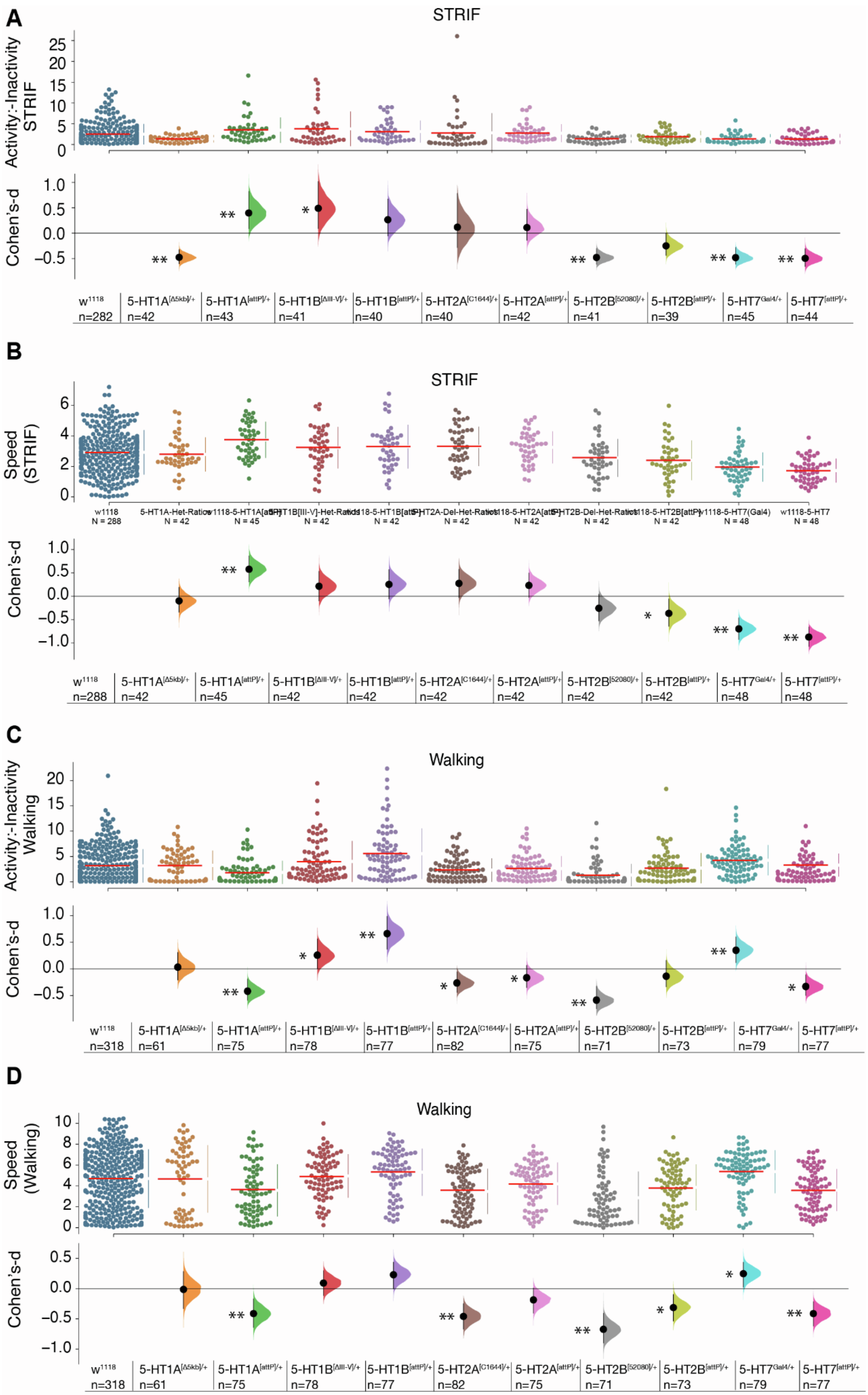
A
STRIF
Activity:-Inactivity STRIF
Cohen's-d
w1118 n=282
5-HT1A[Δ5kb]/+ n=42
5-HT1A[attP]/+ n=43
5-HT1B[ΔIII-V]/+ n=41
5-HT1B[attP]/+ n=40
5-HT2A[C1644]/+ n=40
5-HT2A[attP]/+ n=42
5-HT2B[52080]/+ n=41
5-HT2B[attP]/+ n=39
5-HT7[Gal4]/+ n=45
5-HT7[attP]/+ n=44
B
STRIF
Speed (STRIF)
Cohen's-d
w1118 N = 288
5-HT1A-Het-Ratio N = 42
w1118-5-HT1A[attP] N = 45
5-HT1B[III-V]-Het-Ratio N = 42
w1118-5-HT1B[attP] N = 42
5-HT2A-Del-Het-Ratio N = 42
w1118-5-HT2A[attP] N = 42
5-HT2B-Del-Het-Ratio N = 42
w1118-5-HT2B[attP] N = 42
w1118-5-HT7(Gal4) N = 48
w1118-5-HT7 N = 48
w1118 n=288
5-HT1A[Δ5kb]/+ n=42
5-HT1A[attP]/+ n=45
5-HT1B[ΔIII-V]/+ n=42
5-HT1B[attP]/+ n=42
5-HT2A[C1644]/+ n=42
5-HT2A[attP]/+ n=42
5-HT2B[52080]/+ n=42
5-HT2B[attP]/+ n=42
5-HT7[Gal4]/+ n=48
5-HT7[attP]/+ n=48
C
Walking
Activity:-Inactivity Walking
Cohen's-d
w1118 n=318
5-HT1A[Δ5kb]/+ n=61
5-HT1A[attP]/+ n=75
5-HT1B[ΔIII-V]/+ n=78
5-HT1B[attP]/+ n=77
5-HT2A[C1644]/+ n=82
5-HT2A[attP]/+ n=75
5-HT2B[52080]/+ n=71
5-HT2B[attP]/+ n=73
5-HT7[Gal4]/+ n=79
5-HT7[attP]/+ n=77
D
Walking
Speed (Walking)
Cohen's-d
w1118 n=318
5-HT1A[Δ5kb]/+ n=61
5-HT1A[attP]/+ n=75
5-HT1B[ΔIII-V]/+ n=78
5-HT1B[attP]/+ n=77
5-HT2A[C1644]/+ n=82
5-HT2A[attP]/+ n=75
5-HT2B[52080]/+ n=71
5-HT2B[attP]/+ n=73
5-HT7[Gal4]/+ n=79
5-HT7[attP]/+ n=77

Figure S4. Serotonin receptors affect multiple aspects of STRIF and walking behaviors, Related to Figure 5.

A. Inconsistent effects in STRIF activity-inactivity ratios were observed between two mutant alleles of same receptors, except for 5-HT7 receptors, which show consistently significantly reduced activity-inactivity ratios in both alleles (*5-HT7*$^{[G)}$/*w*^{1118}, n = 45, Δ = -0.455, *d* = -0.482, [95.0%CI -0.611, -0.283], *p = 0.004)* ;(*5-HT7*$^{[attp\}}$ /*w*^{1118}, n = 44, Δ = -0.47, *d* = -0.496, [95.0%CI -0.656, -0.309], *p = 0.003).* ***B.*** Similar to activity-inactivity ratios in STRIF assay, effect on STRIF-speed was variable among receptors, except for 5-HT7, which consistent significantly reduced speed in STRIF paradigm, (*5-HT7*$^{[G)}$/*w*^{1118}, n = 48, Δ = -0.66, *d* = -0.69, [95.0%CI -0.92, -0.46], *p = 0.0)*(*5-HT7*$^{[attp\}}$ /*w*^{1118}, n = 48, Δ = -0.82, *d* = -0.87, [95.0%CI -1.07, -0.65], *p = 0.0).* **C**. In walking paradigm, except for 5-HT2A, two alleles of other receptors show variable results in activity-inactvity ratios (*5-HT2A*$^{[c1644)}$/*w*^{1118}, n = 82, Δ = -0.25, *d* = -0.26, [95.0%CI -0.44, -0.05], *p = 0.02; 5-HT2A*$^{[attP)}$/*w*^{1118}, n = 75, Δ = -0.14, *d* = -0.16, [95.0%CI -0.34, 0.06], *p = 0.19.* ***D.*** In walking paradigm, only 5-HT2B receptor allele show consistent reduced speed, other receptors allele show variable results *5-HT2B*$^{(MI}$/*w*^{1118}, n = 71, Δ = -0.66, *d* = -0.67 [95.0%CI -0.89, -0.40], *p = 0.0; 5-HT2B*$^{(attp)}$/*w*^{1118}, n = 73, Δ = -0.30, *d* = -0.31, [95.0%CI -0.53, -0.09], *p = 0.01)*
